# Sulfur and Phosphorus Oxyacid Radicals

**DOI:** 10.1021/acs.jpca.1c10455

**Published:** 2022-01-27

**Authors:** Michael Bühl, Tallulah Hutson, Alice Missio, John C. Walton

**Affiliations:** EaStCHEM School of Chemistry, University of St. Andrews, St. Andrews, Fife KY16 9ST, U.K.

## Abstract

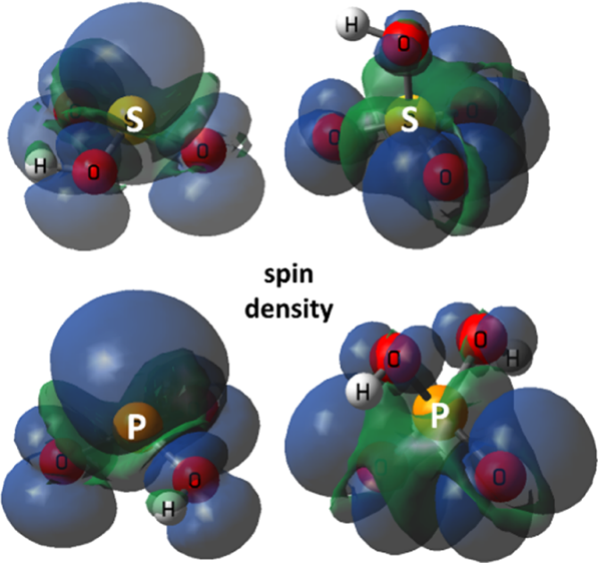

We report a computational study of the little-studied neutral bisulfite, bisulfate, dihydro-phosphite, and dihydro-phosphate radicals (HSO_*x*_^•^, H_2_PO_*x*_^•^, *x* = 3,4), calling special attention to their various tautomeric structures together with p*K*_a_ values estimated from the Gibbs free energies of their dissociations (at the G4 and CAM-B3LYP levels of density functional theory). The energetics of microhydration clusters with up to four water molecules for the S-based species and up to eight water molecules for the P-based species were investigated. The number of microhydrating water molecules needed to induce spontaneous de-protonation is found to correlate the acid strength of each radical. According to the computed Gibbs free reaction and activation energies, S- and P-centered radicals preferentially add to the double bond of propene (a lipid model), whereas the O-centered radical tautomers prefer H-abstraction. The likely downstream reactions of these radicals in biological media are discussed.

## Introduction

**Scheme 1:**
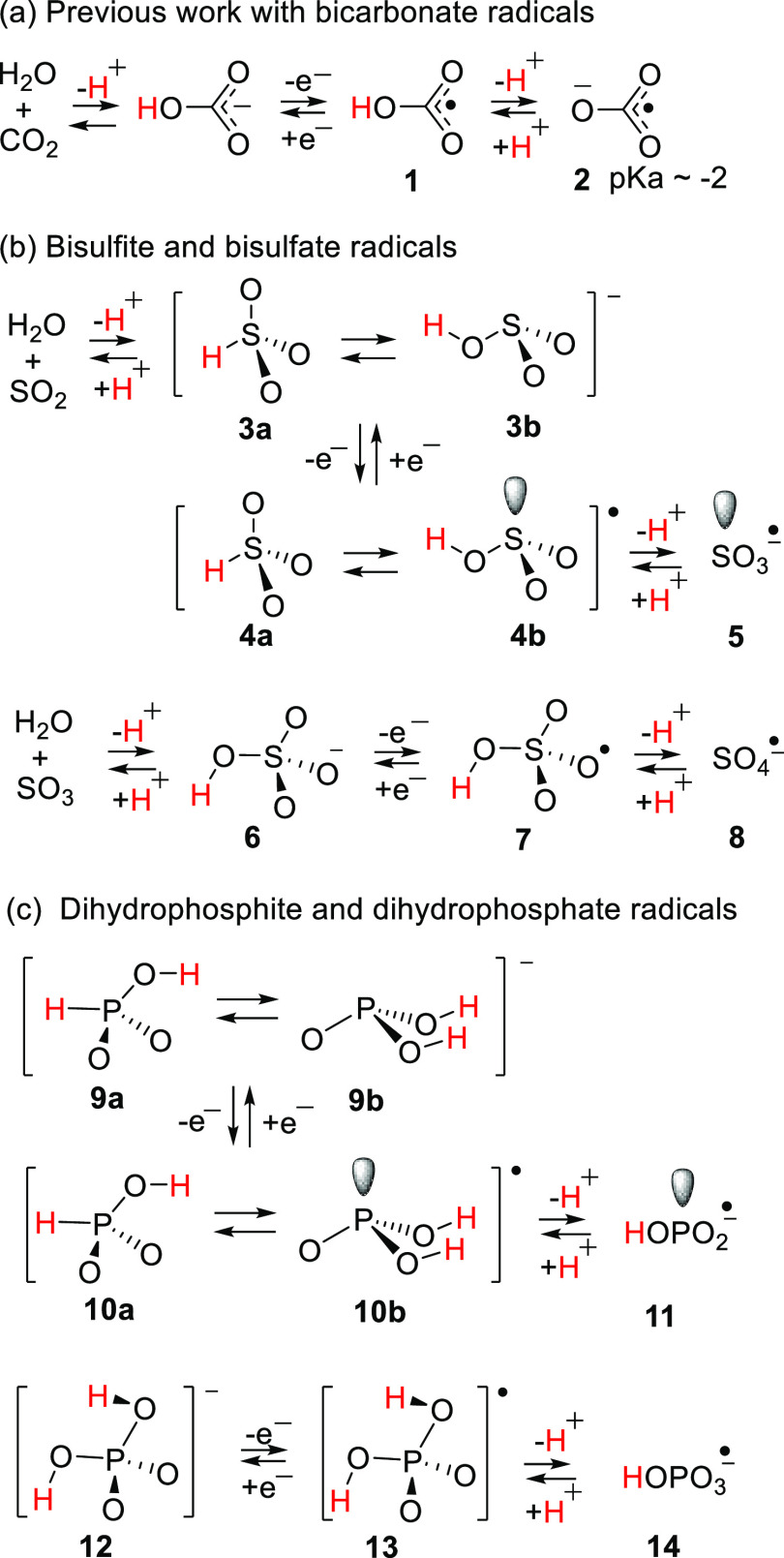
Neutral and Charged Oxy-Radicals of Carbon, Sulfur, and Phosphorus

Although the role of radicals derived from reactive oxygen species (ROS) as a source of oxidative stress in living cells is well established, much less is known about the role of radicals derived from organic or inorganic oxyacids. For example, carbonic, sulfuric/sulfurous, and phosphoric/phosphorous acids are ubiquitous in the biosphere in the form of their conjugate basic anions. Carbonic acid is formed when carbon dioxide dissolves in water, producing a set of species that constitute the bicarbonate buffer system.^1^ Of these, bicarbonate and carbonate play important roles not only in physiology but also in atmospheric chemistry and geology. In living organisms, several enzyme systems oxidize bicarbonate and carbonate to neutral bicarbonate radicals **1** and/or carbonate radical anions **2** (Scheme 1).^2−9^ The bicarbonate radical is a strong acid,^10−12^ with a p*K*_a_ of probably about −2 units,^13^ and so in aqueous solution, the main species is the carbonate radical anion. This is known to contribute to oxidative stress by reacting with polar species such as bio-thiols, nucleic acids, metalloproteins/proteins, and glutathione.^14,15^ Comparatively little is known about the neutral bicarbonate radical, but it may be an important species in hydrophobic environments. Interestingly, studies have indicated that its reactivity differs from that of other ROS such as the hydroxyl radical which mainly abstracts H-atoms from unsaturated lipids. An experimental study with model MeCO_2_^•^ radicals showed that these preferentially add to oleate and linoleate double bonds.^11^

Oxy-radicals derived from sulfur and phosphorus are also important in living systems, but the neutral types have received very little study. Sulfur dioxide is a harmful pollutant that readily dissolves in water forming bisulfite (HSO_3_^–^, **3a,b**) and sulfite (SO_3_^2–^). Sulfite and bisulfite compounds are routinely used as preservatives and antioxidants in food and drinks.^16,17^ Sulfite toxicity can cause allergic reactions, asthma, and anaphylactic shock in some cases. In the body, sulfite species are detoxified by molybdenum-dependent sulfite oxidase enzymes which oxidize them up to their corresponding sulfates.^18,19^ Some recent studies have suggested that the toxicity of the bisulfite species is due to their increasing concentration of ROS.^20^ Bisulfite can also undergo autoxidation by transition metal catalysts (Cu^2+^ or Fe^3+^)^21,22^ with formation of the sulfite anion radical SO_3_^–•^ (**5**). The sulfate anion radical SO_4_^–•^ (**8**) is known to cause detrimental protein oxidation by abstracting hydrogen atoms and forming protein radicals.^23^

Phosphates are ubiquitous biological molecules and play vital roles in cellular metabolism.^24,25^ They are a key unit in the backbones of DNA and RNA, the carriers of genetic information. Adenosine monophosphate, adenosine triphosphate (ATP), and analogues are drivers of many biological processes. Up to 70% of bone is composed of a modified form of hydroxyapatite [Ca_10_(PO_4_)_6_(OH)_2_] which also occurs in teeth. The archetype compound is phosphoric acid, which dissociates into dihydrogen phosphate (**12**) and hydrogen phosphate. Single electron oxidation produces the radical species H_2_PO_4_^•^ (**13**) and HPO_4_^– •^ (**14**). Similarly, radicals with the general formula RO(HO)PO_2_^•^ can be created from phosphate esters like AMP and ATP where R is the sugar-based unit. Dihydrogen phosphite **9a,b** can be oxidized to corresponding dihydrogen phosphite radicals **10a,b,** and acid dissociation produces hydrogen phosphite radical anion **11** as a conjugate base (Scheme 1). It is known that phosphate radicals participate in biological processes,^26,27^ but the main structures are unknown, and research on their reactivities is very limited.

These oxy-radicals of sulfur and phosphorus undoubtedly play important roles in living organisms and in the environment. Hence, knowledge about their properties and reactivities is very desirable. Because of their acidity, neutral sulfur-based radicals **4** and **7** and phosphorus-based radicals **10** and **13** are difficult to study experimentally. We therefore adopted a computational approach, similar to that which had proved successful for bicarbonate radicals.^11,28^ Experimental work with laser flash photolysis and pulse radiolysis methods, mainly from the 1960s and 70s, had shown that free radicals were often more acidic than their non-radical precursors.^29^ Subsequent ab initio computations by Radom and co-workers on radicals of the type ^•^CH_2_X had supported this conclusion for C-centered radicals.^30−34^ It is now known that radicals centered on C-, N-, and O-atoms all enhance acidity and that this increases with the electronegativity of the radical center. Experimental and computational research on radical acidity was reviewed recently.^35^ It seemed likely, therefore, that neutral sulfur-based radicals **4** and **7** and phosphorus-based radicals **10** and **13** would also be strong acids. Indeed, a previous computational study on the sulfinic radicals HOSO_2_^•^ (**4b**) had confirmed this.^36^ The present research was undertaken to probe the structures, acidities, and likely reactions with lipid components of oxy-radicals based on sulfur and phosphorus and to compare them with carbon-based analogues.

## Computational Details

Ab initio and DFT calculations were carried out using the Gaussian 09 suite of programs.^37^ Based on benchmark calculations against the highly accurate Gaussian-4 (G4)^38^ method (see the Supporting Information for details), the CAM-B3LYP functional^39^ which combines the hybrid qualities of B3LYP with the long-range correction proposed by Tawada et al.^40^ was confirmed as a functional of choice for this study. Geometries were optimized at that level using the 6-31G(d,p) basis set, followed by single-point energy (SPE) calculations on the pre-optimized structures using the 6-311+G(2d,p) basis set unless otherwise specified. In selected cases, full optimizations were carried out at the CAM-B3LYP/6-311+G(2d,p) level. Solvent effects were accounted for with the CPCM continuum model,^41^ except as otherwise indicated. Default values of the keywords Alpha, Radii, TSNUM, and TSARE were employed. Open-shell systems were treated with the spin-unrestricted Kohn–Sham formalism. Vibrational frequency calculations were performed at the same level as the geometry optimizations and were used to characterize minima (no imaginary frequencies) and transition states (one imaginary frequency) and evaluate enthalpies and free energies through the zero point and thermal corrections at 1 atm and 298 K. The lowest energy structures of conformationally flexible molecules were, in most cases, initially estimated from related molecules computed previously. For the isolated molecules in the gas phase, G4 data are discussed preferentially, while for the acidities and reactivities of the radicals, the CAM-B3LYP levels detailed above were used.

## Results and Discussion

### Structures of the Sulfur and Phosphorus Oxy-Radicals

**Chart 1:**
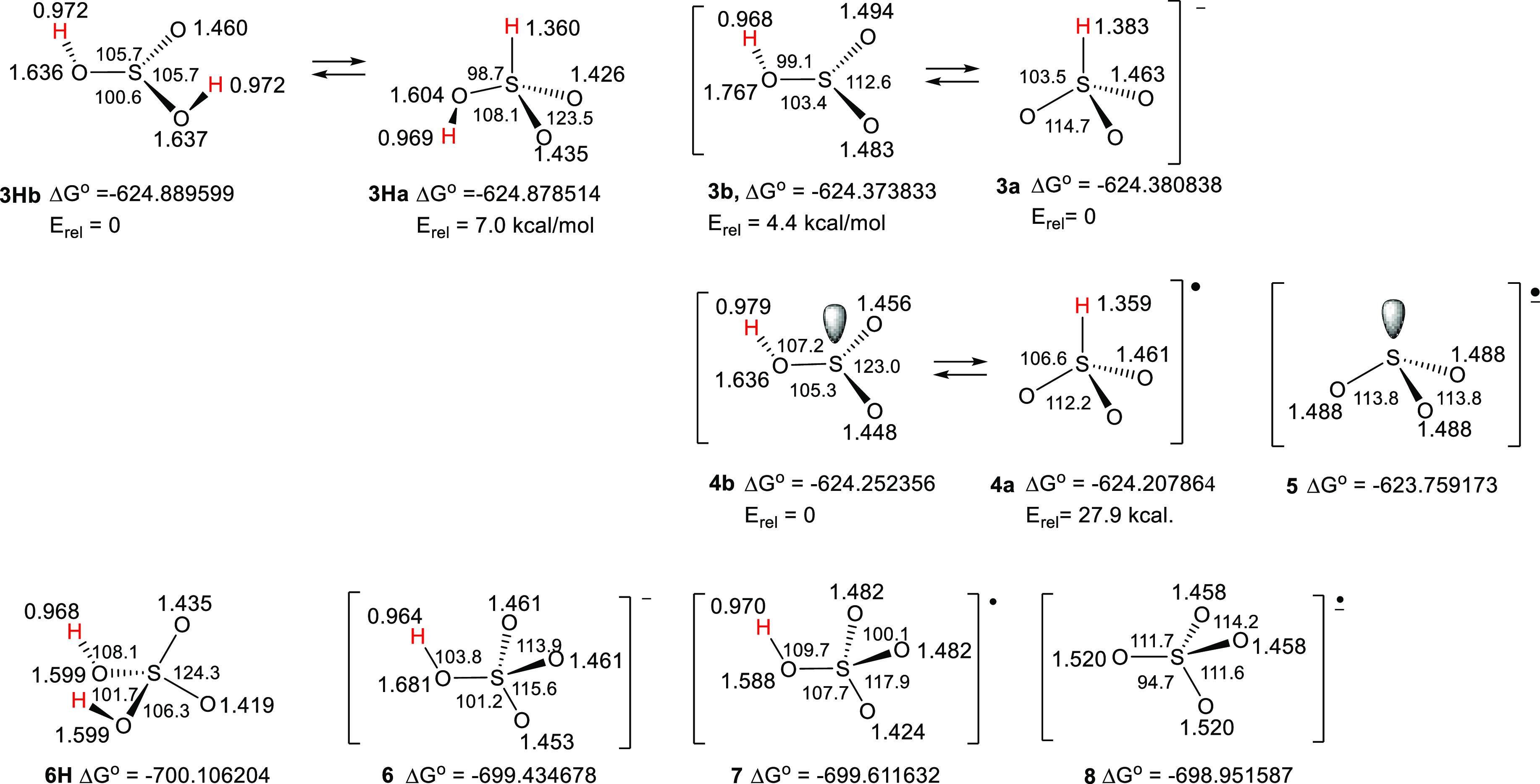
Structures of sulfur oxy-radicals, anions, and anion radicals, including absolute (Δ*G*^o^, in a.u.) and relative free energies (*E*_rel_, in kcal/mol) at the G4 level and selected bond distances and angles in Å and degrees, respectively [at the same level, i.e., B3LYP/6-31G(2df,p) optimized].

The optimized structures of bisulfite radicals **4a** and **4b** and bisulfate radical **7** plus the structures of the associated acids, anions (**3a, 3b, 6**), and anion radicals (**5**, **8**) were obtained at the G4 level in vacuum, and key parameters are illustrated in Chart 1.

For each of the precursor sulfurous acids (**3Ha**, **3Hb**), anions (**3a**, **3b**), and radicals (**4a**, **4b**), two isomeric (tautomeric) structures exist. The “**a**” structures contain S–H bonds, and the “**b** structures contain only O–H bonds. The two isomers of acid **3Ha,b** and of anion **3a,b** were computed to be comparatively close to each other in energy, but S–H-containing isomer **4a** of the bisulfite radical was 27.9 kcal/mol higher in energy than the **4b** isomer. Accordingly, it was concluded that the latter was the dominant species, and its properties and reactivity were further investigated. For bisulfate radical **7,** computations indicated that the isomer with a S–H bond (and five coordination about S, not shown) did not have a stable ground state (an attempted optimization resulted in the H moving to the nearest O atom affording **7**). As expected, anions **3a**, radicals **4a,** and anion radicals **5** had C_3v_ symmetry. In comparison, anions **3b** and radicals **4b** had unsymmetrical pyramidal structures. The S–O bond lengths of radical **4b** were somewhat shorter than those of model acid **3Hb,** and the bond angles of **4b** indicated a shallower pyramid in comparison to **3Hb**. Bisulfate radical **7** was found to possess a mirror plane.

Interestingly, the computations indicated that dominant bisulfite radical **4b** and conjugate anion radical **5** had much spin centered of their S-atoms and were essentially sulfur-centered species. By way of contrast, bisulfate radical **7** and conjugate anion radical **8** were essentially oxygen-centered species. The computed EPR hyperfine splittings (hfss) are compared in Table 1 with those obtained experimentally.^42,43^ The large magnitude of the computed *a*(^33^S) values for bisulfite radical **4b** and sulfite radical anion **5** supports the conclusion that these species were substantially sulfur-centered. This was confirmed in the case of **5** by the experimental *a*(^33^S) (Table 1) that was in reasonable agreement with the computed data. The EPR spectrum of bisulfite radical **4b** was observed, but the hfs of the minor isotope ^33^S (0.75% natural abundance) was not obtained.^44^ The comparatively small *a*(^33^S) values for bisulfate radical **7** and sulfate anion radical **8** supported the conclusion that these were predominantly O-centered intermediates. The sulfate anion radical has been detected many times by EPR spectroscopy, and the ^33^S hfs was unresolved as would be expected from the small computed value.

**Table 1:** Computed and Experimental EPR Parameters of Sulfur and Phosphorus Oxy-Radicals^a^^,^^b^

radical	structure	method	*a*(^33^S)	*a*(H)–S	*a*(H)–O	*a*(^17^O)	*a*(^17^O)	*a*(^17^O)–H
**4b**	^•^SO_2_OH	comp.	116.0		–3.8	–4.6,-3.7		–13.0
**4a**	HSO_3_^•^	comp.	–7.0	–3.2		–1.3 (x3)		
**5**	^•^SO_3_^–^	comp.	97.0			–10.5(x3)		
**5**	^•^SO_3_^–^	expt.	(±)102					
**7**	HOSO_3_^•^	comp.	–8.3		–0.3	–3.6 (x2)	0.1	1.3
**8**	SO_4_^–•^	comp.	–6.6			–6.1 (x2)	0.5 (x2)	
**8**	SO_4_^–•^	expt.	n.r.^c^					

radical	structure	method	*a*(^31^P)	*a*(H)–P	*a*(H)–O	*a*(^17^O)	*a*(^17^O)	*a*(^17^O)–H
**10a**	HPO_2_^•^OH	comp.	–50.5	–2.7	–1.2	–2.3 (x2)		1.8
**10b**	^•^PO(OH)_2_	comp.	713.9		–2.1, −3.2	–6.2		–17.3,-19.7
**11b**	^•^PO_2_OH^–^	comp.	561.3		–1.0	–13.3 (x2)		–28.1
**11b**	^•^PO_2_OH^–^	expt.	(±)658		n.r.			
	^•^PO(OMe)_2_	expt.	(±)685					
	^•^PO(OMe)O^–^	expt.	(±)556					
**13**	(HO)_2_PO_2_^•^	comp	–51.2		–0.5, −0.8	–3.6 (x2)		1.4,1.0
**14**	HOPO_3_^–•^	comp.	–40.5		–0.2	–4.8 (x2)	–0.5	1.3
**14**	HOPO_3_^–•^	expt.	(±)34.5		n.r.			

a Computed at the UCAM-B3LYP/6-311+G(2d,p)//B3LYP/6-31G(2df,p) level in vacuum.

b Hyperfine splittings in Gauss.

c n.r. = not resolved.

**Chart 2:**
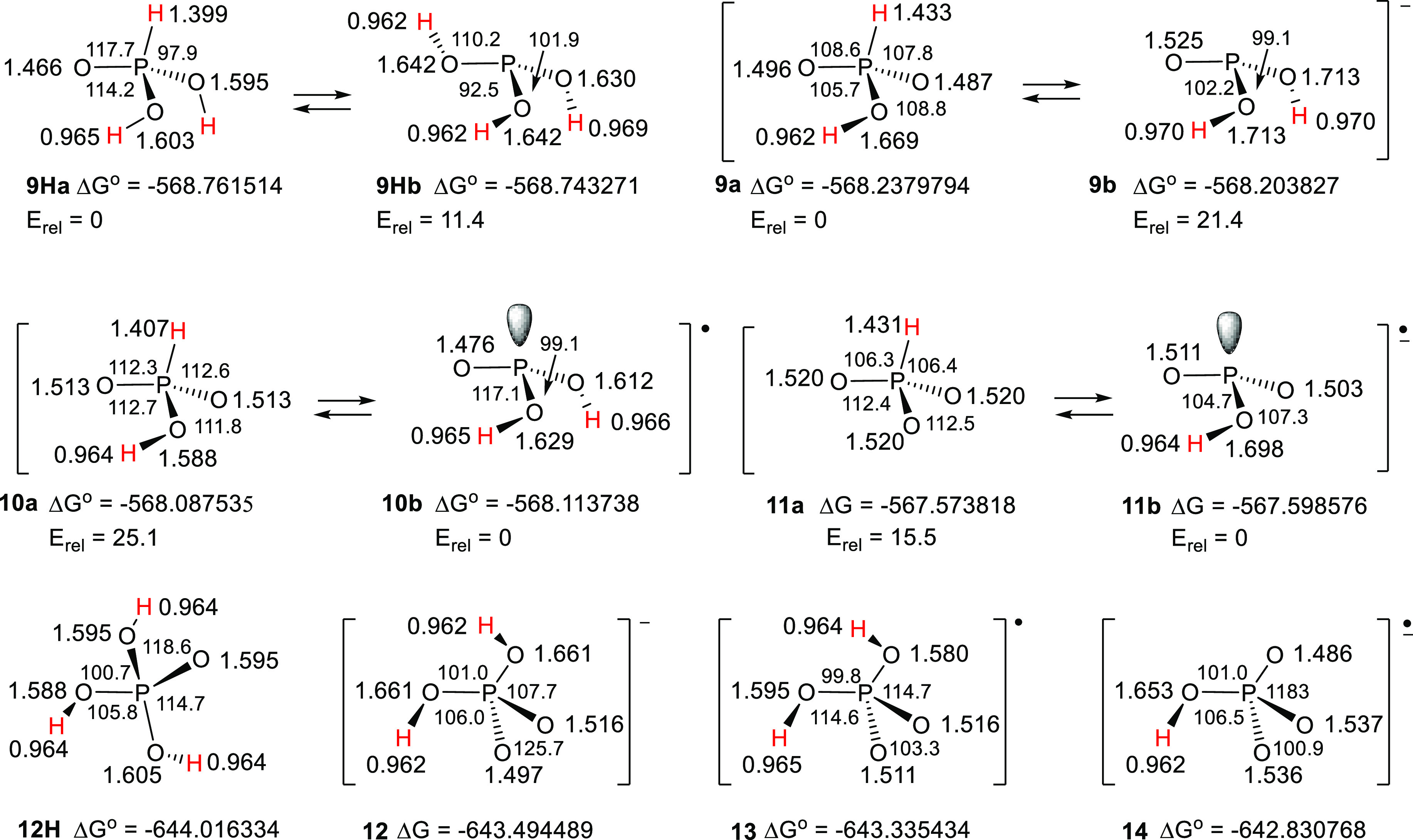
Structures of phosphorus oxy-radicals, anions, and anion radicals.

The G4 (i.e., B3LYP)-optimized structures of phosphonyl radicals **10a** and **10b**, (dihydrophosphite radicals), and the dihydrophosphate radical (**13**) plus the structures of the associated acids and anion radicals are illustrated in Chart 2.

The computations indicated that P-centered isomer **10b** was 25.1 kcal/mol lower in energy than isomer **10a** and hence would be the predominant species. Similarly, conjugate anion radical **11a** was found to be 15.5 kcal/mol higher in energy than tautomer **11b**. Unsymmetrical pyramidal structures were found for both radical **10b** and its conjugate anion radical **11b,** and both were shallower than model acid **9Hb**. Anion radical **14** contained a mirror plane. The large computed *a*(^31^P) values for **10b** and **11b** showed that significant spin was associated with their P-atoms, in keeping with the analogous S-radicals discussed above. The EPR spectrum of neutral radical **10b** has not been observed, but its computed *a*(^31^P) is close to that of the model neutral radical ^•^PO(OMe)_2_ (Table 1).^45^ The computed hfs of **11b** was in reasonable agreement with the EPR experimental value and that of model species ^•^PO(OMe)O^–^ (Table 1). The comparatively small computed *a*(^31^P) values for dihydrophosphate radical **13** and conjugate anion radical **14** indicated that they were predominantly O-centered species. The experimental EPR spectrum of **13** has not been observed. The experimental *a*(^31^P) for **14** was small in magnitude, also in reasonable agreement with the computed value (Table 1).

### Acidities of the Oxy-Radicals of Sulfur and Phosphorus

Whether the above-mentioned oxy-radicals react in their neutral or anionic forms depends on their p*K*_a_s. The properties of the neutral and anionic forms are expected to be very different because the extent of electron delocalization differs, and the presence of the charge will strongly enhance hydrophilicity. Physiologically, the anion radicals will tend to reside in aqueous milieus, whereas the neutral forms will prefer non-polar, lipid environments. The p*K*_a_s are difficult to determine experimentally because of the transient nature of the radicals. We therefore used a computational method to estimate these quantities. The p*K*_a_ of a Bronsted acid HA is usually proportional to the free energy of deprotonation Δ*G*_HA_, and hence, computing these gives a way of accessing the acidities. As explained below, direct calculation of a p*K*_a_ from a computed Δ*G*_HA_ is challenging. A more practical, fruitful method relies on obtaining DFT-computed Δ*G*_HA_s for sets of acids with known p*K*_a_s. The acidities of unknown species can then be obtained from the resulting simple linear regression (SLR) line.^46,47^ In order to make the estimates as reliable as possible, molecules with structures similar to the unknown species were chosen for the model study. The range of suitable acids with known p*K*_a_s is limited, but a group of nine phosphorus-based acids together with six sulfur-based acids was exploited to establish a linear correlation (Table 2). This allowed for a direct comparison between the sulfur and phosphate radicals. The calculations were carried out with the aqueous phase modeled using the CPCM continuum. For comparison purposes, similar computations were carried out in vacuum. Free energies can be computed for the deprotonation process (Δ*G*_HA_) eq 1
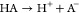
1and for the proton transfer to water (Δ*G*_HA/H_2_O_) eq 2
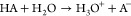
2

**Table 2:** Computed Free Energies of Proton Transfer for P-Based and S-Based Acids with Known p*K*_a_s

acid	Δ*G*_HA/H_2_O_ (vac) kcal/mol	Δ*G*_HA/H_2_O_ (aqu) kcal/mol	p*K*_a_ (exptl.)^a^
H_3_PO_4_	319.6	9.1	2.16
HPO(OH)_2_	320.7	9.2	1.5
P(OH)_3_	330.3	18.4	7.4
P(OH)_2_(OEt)	332.3	19.2	6.7
HPO(OH) (OEt)	328.3	9	0.9
P(OH)(OEt)_2_	332.1	22.2	6.1
HPO(OEt)_2_	342.3	34.6	13
PO(OH)(Et)_2_	329.9	19	3.08
PO(OH)(^*t*^Bu)_2_	326.5	20.9	6.08
H_2_SO_4_	301.9	–7.6	–3
H_2_SO_3_	159.6	4.7	1.82
HSO_3_Me	315.0	–1.9	–0.6
HSO_3_F	290.2	–17.2	–10.5
HSO_3_Et	310.8	–0.26	–1.7
HSO_3_CF_3_	291.1	–14.1	–12

a Experimental p*K*_a_ data as follows: H_3_PO_4_,^49^ HPO(OH)_2_, P(OH)_3_, P(OH)_2_(OEt), HPO(OH)(OEt), P(OH)(OEt)_2_ and HPO(OEt)_2,_^50^ PO(OH)(Et)_2_,^51^ PO(OH)(^*t*^Bu)_2_,^52^ H_2_SO_4_ and HSO_3_Me,^53^ H_2_SO_3_,^54^ HSO_3_F,^55^ HSO_3_Et,^56^ and HSO_3_CF_3_,.^57^

The free energy of solvation for the proton cannot be easily computed quantum-chemically, but from the known literature data, we chose the fairly recent value of −264.2 kcal/mol obtained by Tissandier et al.^48^ In fact, the Δ*G*_HA_ and Δ*G*_HA/H_2_O_ values from the two approaches simply differ by a constant {[Δ*G*(H_3_O^+^) – Δ*G*(H_2_O)] – 264.2}, so there is no advantage of one approach over the other. The computed Δ*G*_HA/H_2_O_ data in aqueous and vacuum phases are set out in Table 2 for the set of acids.

**Figure 1:**
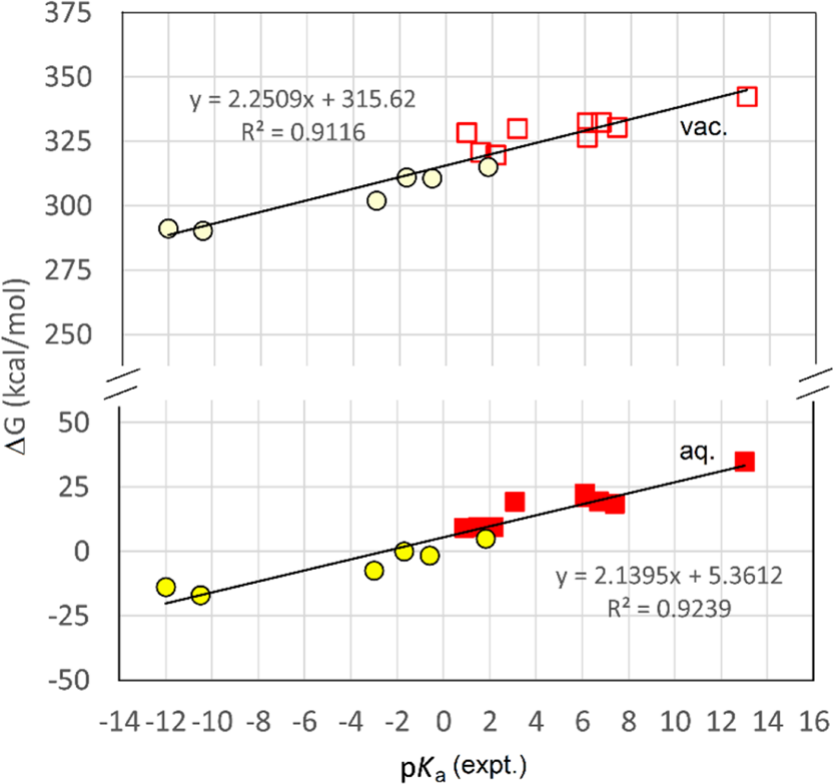
SLR plots of computed free energies for the acids of Table 2 with known p*K*_a_s. Upper line; computations in vacuum. Lower line; computations in the aqueous phase. Squares represent P-based acids, and circles correspond to S-based acids.

Satisfactory linear SLR correlations were obtained from both sets of data (Figure 1), and the following relationships resulted
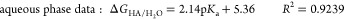
3
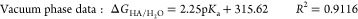
4

The Δ*G*_HA/H_2_O_ values for proton transfer of the S- and P-based radicals in aqueous and vacuum phases were computed with the same DFT method (Table 3).

**Table 3:** Free Energies and Derived p*K*_a_s for Radical Intermediates^a^

radical	Δ*G*_HA/H_2_O_ (vac.) kcal/mol	Δ*G*_HA/H_2_O_ (aq.) kcal/mol	p*K*_a_ (vac.)	p*K*_*a*_ (aq.)	Δp*K*_a_^c^
^•^SO_2_OH, **4b**	301.4	–6.2	–6.33	–6.71	8.5
HOSO_3_^•^, **7**	293.6	–9.6	–9.77	–9.25	6.3
^•^PO(OH)_2_, **10b**	307.9	–3.3	–3.43	–2.92	4.84
HPO_2_^•^OH, **10a**	316.3	0.5	0.30	0.19	1.3
(HO)_2_PO_2_^•^, **13**^·^	309.2	–2.7	–2.83	–2.27	4.4
HOCO_2_^•^^b^				–1.16	4.8

a Free energies in kcal/mol.

b Data from ref (26).

c Difference between the p*K*_a_s of the model acids and the p*K*_a_(aq.) data of the radicals, computed at the same level of theory.

For each species, the p*K*_*a*_ s computed from the vacuum and aqueous data agree to within ∼0.6 units which lends confidence to the reliability of the results. The bisulfite (**4b**) and bisulfate (**7**) radicals were both found to be very strong acids. As expected from the greater extent of electron delocalization in its conjugate base, **7** was an even stronger acid, being almost in the super-acid class. The acidities of the dihydrogen phosphite (**10b**) and dihydrogen phosphate (**13**) radicals were similar to each other, but they were both much weaker acids than bisulfite and bisulfate radicals, as expected from the greater electronegativity of sulfur. Tautomer **10a** is much higher in energy than tautomer **10b** and therefore converts to this prior to proton loss. The conjugate base of both tautomers will be **11b,** and the p*K*_a_s were evaluated on this basis. Favored dihydrogen phosphite radical isomer **10b** was significantly more acidic than less favored isomer **10a**.

It is noteworthy that the S-based and P-based radicals were all significantly more acidic than the model acids in which the unpaired electron (upe) was replaced by a H-atom. The last column of Table 3 shows the differences of the radical p*K*_a_s from those of the model acids: Δp*K*_a_ = p*K*_a_(model) – p*K*_a_(radical). Just as was found with the bicarbonate radical (bottom row) and other radicals based on first-row elements,^33^ the S- and P-based radicals are 4 or more orders of magnitude more acidic than the parent acids (except for disfavored tautomer **10a**). This is likely due to the extra thermodynamic stabilization of the conjugate anion radicals released on deprotonation of the radicals as compared to the closed-shell anions released from the parent acids. A possible rationale is that after removal of a proton from a OH group, the resulting terminal O atom is not only available for delocalization of the negative charge but also the upe.

### Microhydration of the S- and P-Based Oxy-Radicals

That solvation will play a key role in the stabilization of the conjugate anion radicals is beyond question. Charge and spin will be distributed unevenly in these species depending on their topological details. However, the DFT computations employed the CPCM continuum model that provides an averaged electrostatic reaction field and neglects specific solute–solvent interactions. This model could well give an inadequate description of solvation for the small charged anion radicals of this study. Previous experimental and theoretical research had disclosed that mineral acids HA spontaneously ionize when associated with only a few microsolvating water molecules to afford ion pairs: [A^–^(H_2_O)_*n*−1_(H_3_O^+^)].^58,59^ A tendency for stronger acids to require fewer solvating water molecules to induce ionization was noted.^60,61^ This trend presented an independent way of checking the high acidities computed for radicals **4b**, **7**, **10b,** and **13**. We therefore investigated the effect of successively expanding solvating water clusters around these species. For the S-based radicals, hydrated cluster configurations [HOSO_*x*_^•^*n*H_2_O] were investigated, at the CAM-B3LYP/6-311+G(2d,p) level, for *n* = 1 to 4. In each case, the effect of the bulk solvent was represented with the CPCM continuum model. For the P-based radicals, hydrated cluster configurations [(HO)_2_PO_*x*_^•^*n*H_2_O] were investigated with the same DFT method for *n* = 1 to 9.

The number of possible 3D configurations for each cluster increases steeply as the number of hydrating water molecules increases. Identifying the true global minima is difficult because the potential energy surfaces become complex and contain many shallow local minima. Microhydration structures for formic and trifluoroacetic acid were published by Maity,^62,63^ and Leopold reviewed microhydration for a set of mineral acids.^57^ These examples, together with the optimum cluster structures established for a set of hydrated carboxylic acid radicals,^59^ enabled some useful rules for identifying the lowest energy clusters to be established. For each value of “*n*”, the lowest energy structure of the acid radical cluster normally contains the following: (i) a water molecule H-bonded to the acidic proton(s) of the substrate, (ii) maximum possible H-bonds to the O-atoms of the acid, (iii) the maximum number of H-bonds to each water molecule, and (iv) five- and six-member rings of O-atoms (or other heavy atoms) rather than chains or dangling water molecules. By applying these criteria, it was possible to limit to a few the number of cluster configurations that needed to be investigated for each of the radicals.

**Figure 2:**
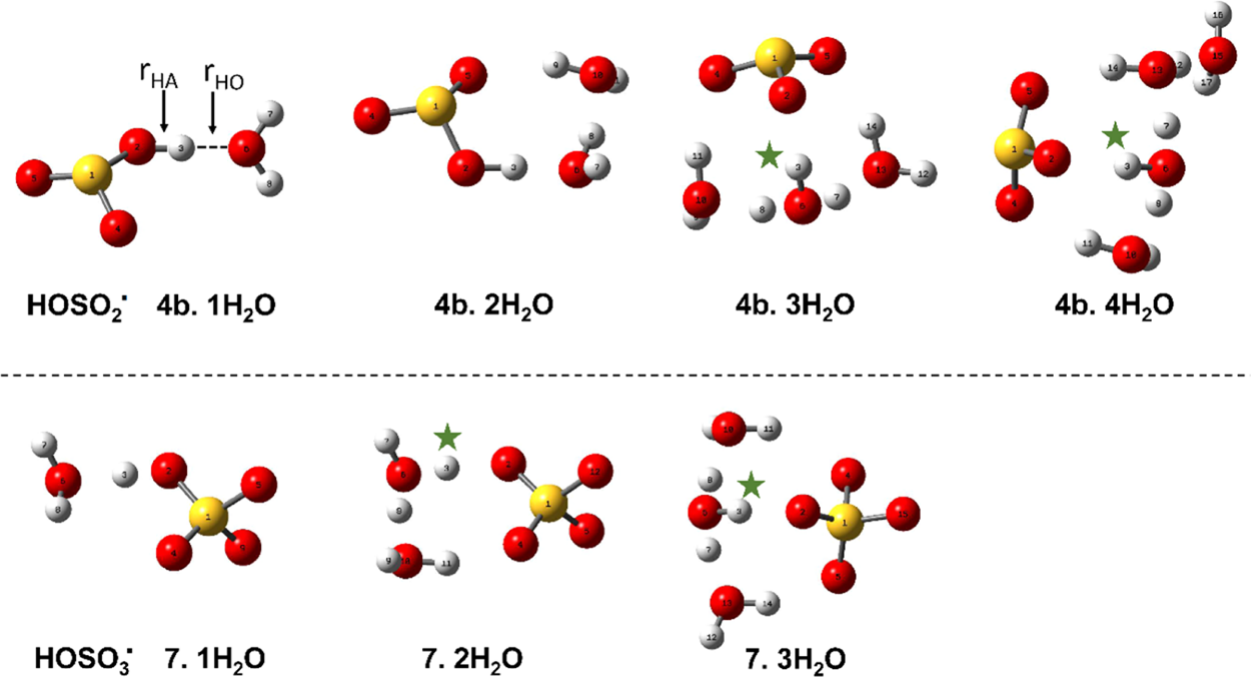
Optimized cluster structures for bisulfite (**4b**) and bisulfate (**7**) radicals with increasing hydration. The dissociated protons are marked with green asterisks.

For the bisulfite and bisulfate radicals, a series of initial structures was tested for each value of “*n*”. The lowest energy structures (which should be representatives of the global minima) associated with radical **4b** for *n* = 1 to 4 and for radical **7** with *n* = 1 to 3 H_2_O molecules are displayed in Figure 2.

**Figure 3:**
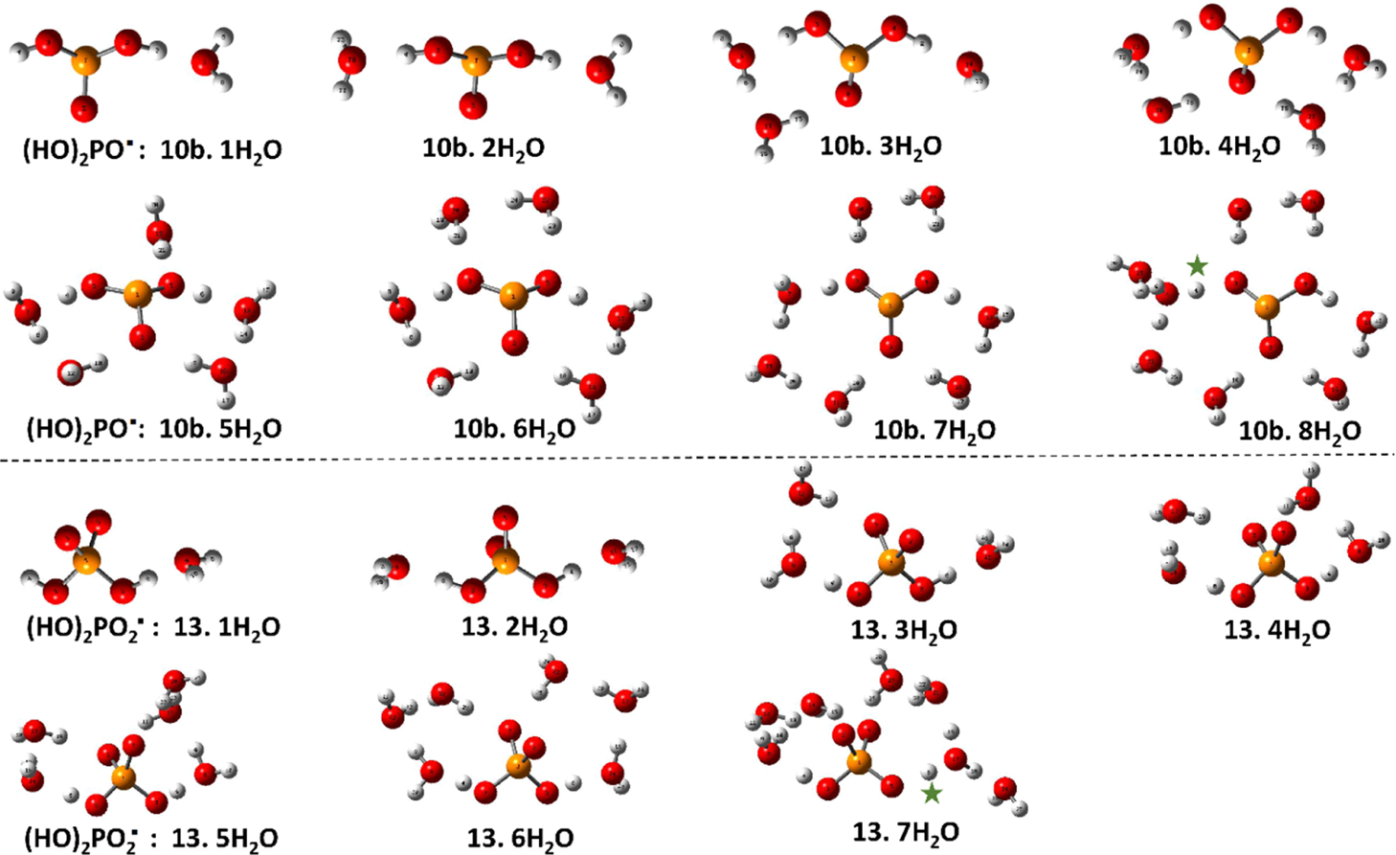
Optimized cluster structures for dihydrogen phosphite (**10b**) and dihydrogen phosphate (**13**) radicals with increasing hydration. Dissociated protons are marked with green asterisks.

Similarly, the global minimum energy structures associated with phosphite radical **10b** for *n* = 1 to 8 and for phosphate radical **13** with *n* = 1 to 7 H_2_O molecules are shown in Figure 3. In conformity with the criteria listed above, these optimized clusters all have H-bonds from water to the acidic H-atoms plus rings or 3D cages of five heavy atoms that maximize the number of H-bonds among the water molecules. Minimum dangling water molecules were observed. In the case of di-hydric P-radicals **10b** and **13,** initial structures in which the water molecules were clustered on one side, leaving one acidic H-atom without a H-bond, were tried. However, in each case, these structures were higher in energy than those illustrated which contained H-bonds to both acidic H-atoms. For clarity, the ionizing proton is marked with a green asterisk.

That the structures shown were the global minima or close representatives was supported by plots of their Δ*G*° values against the number of water molecules involved (see Figure S1 in the Supporting Information). These were all good straight lines for all four series of clusters (S and P) each having the same slope (−76.43 ± 0.01 kcal mol ^–1^ per unit H_2_O) as expected for species incremented by one water molecule.

**Figure 4:**
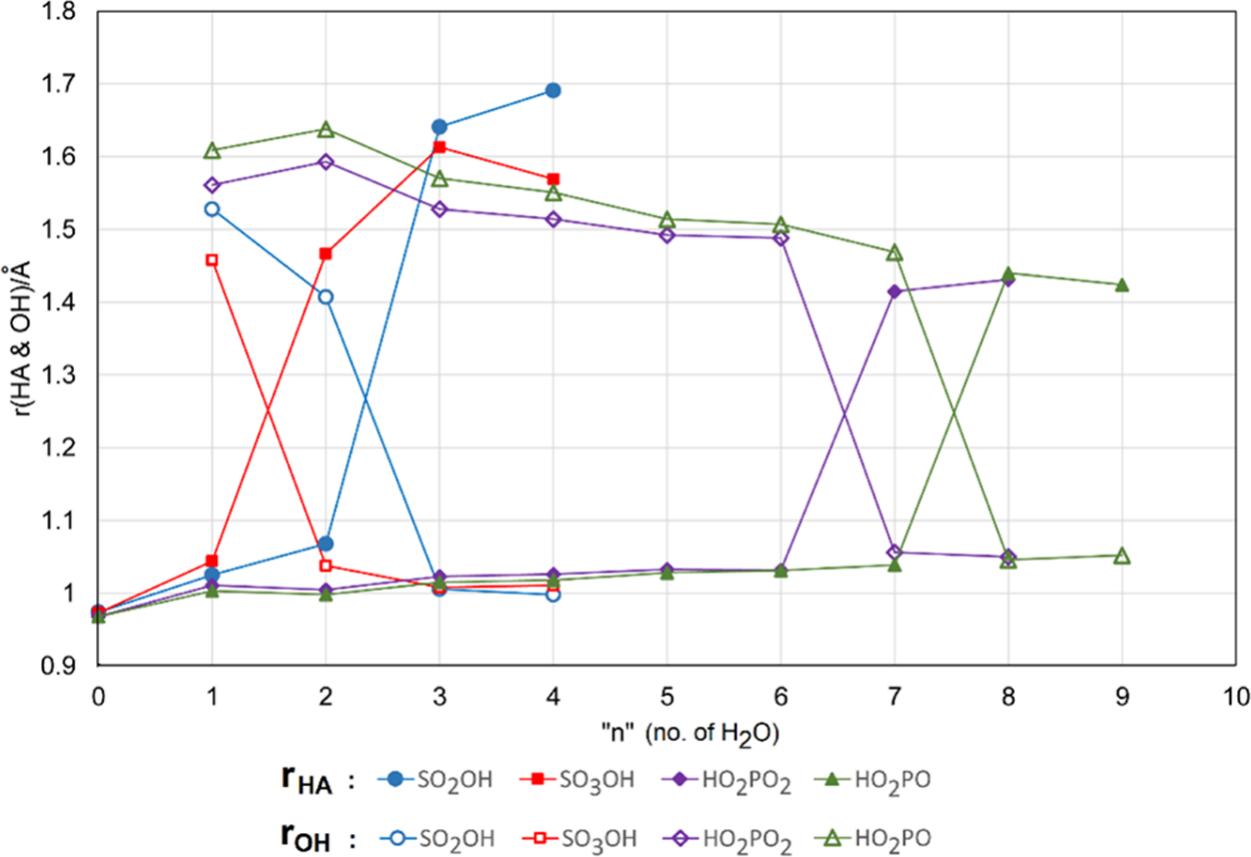
Computed lengths of the radical A–H bonds *r*_HA_ and the lengths of the bonds from the leaving proton to the nearest H_2_O molecule, *r*_HO_, against the number of water molecules in each cluster.

For each series of clusters, the computed lengths of the radical A–H bonds *r*_HA_ and the lengths of the bonds from the leaving proton to the nearest H_2_O molecule, *r*_HO_, were useful indicators of the state of ionization. These distances are plotted in Figure 4 as a function of the number of H_2_O molecules in the global minimum clusters for radicals **4b, 7, 10b,** and **13**. For each radical, the *r*_AH_ distance remained close to 1.0 Å with increasing hydration until, at ionization, a steep increase to the range 1.4–1.6 Å took place. This pattern was essentially mirrored by the *r*_HO_ distances which were in the range 1.4–1.6 Å for unionized clusters and steeply decreased to about 1.0 Å for the ionized clusters.

Remarkably, spontaneous ionization took place for bisulfate radical **7** when associated with only *n* = 2 water molecules. Similarly, ionization of bisulfite radical **4b** occurred for only *n* = 3 microhydrating water molecules. Thus, fewer water molecules were needed to induce ionization than with mineral acids like H_2_SO_4_ and HCl, which is in good agreement with the very negative p*K*_a_ values computed for these S-based radicals.

**Figure 5:**
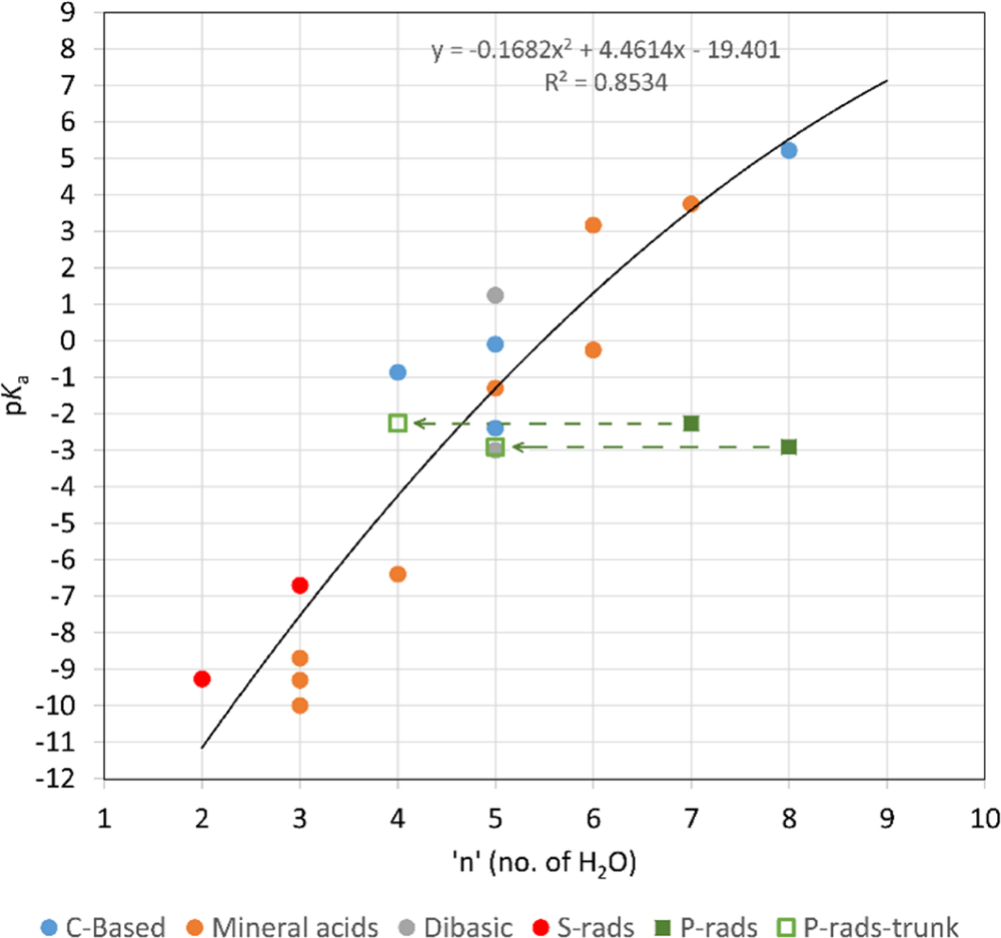
Graph of the number of water molecules (n) required to induce ionization against the p*K*_a_ for a selection of mineral and radical acids. Blue circles: carboxylic acid radicals, orange and gray circles: mineral acids, red circles: S-based radicals, green squares: P-based radicals, and open green squares: “truncated” clusters with fewer water molecules that are not the most stable configurations, see text.

Much larger clusters were needed to induce ionization of the dihydric P-based acid radicals. Figure 4 demonstrates that *n* = 7 water molecules were required by radical **13** and *n* = 8 water molecules by radical **10b**. Qualitatively, this supports the much weaker acidity of these radicals compared to the S-based radicals. The p*K*_a_s of dihydrogen phosphite radical **10b** and dihydrogen phosphate radical **13** were scarcely distinguishable (Table 3), but slightly stronger **10b** actually required one more microsolvating water molecule to induce ionization than **13.** The plot in Figure 5 of the p*K*_*a*_ values against the computed microsolvating “*n*” values for these second-row acid radicals compares them with previous data for mineral acids and carboxylic acids.

The mineral, carboxylic, and S-based acids show a general trend of decreasing “*n*” as the acidity of the species increases. However, the two P-based radicals (filled green squares in Figure 5) were anomalous in needing more water molecules for ionization than suggested by the general trend. It is likely that this is related to their dihydric nature. To minimize the cluster energies, H-bonds from water molecules to both the OH groups of the (HO)_2_PO_*x*_^•^ radicals were required (see above). A consequence of this was that the total number of water molecules required for the first proton ionization was inflated by those H-bonding to the non-ionizing HO group of the radical. This factor increased the “*n*” values of these dihydric species in comparison with those of the monohydric mineral and carboxylic acid species. If only the water molecules forming the hydration shells around the ionizing protons are included, the “*n*” values for phosphite **10b** and phosphate **13** radicals reduce to five and four, respectively. These values fall within the region of the correlation (see arrowed markers in Figure 5) which is not strictly linear but can be fitted, without implying an underlying physical reason, with a second-order polynomial. The ionized conjugate radical anions will be more strongly hydrated than the neutral radical precursors. It is plausible, therefore, that higher proton transfer energies will require more water molecules to overcome this. The general correlation of “*n*” with the computed p*K*_a_ values demonstrates the validity of this relationship. That the correlation is non-linear and somewhat scattered is to be expected from the diversity of the species involved.

### Addition and Abstraction Reactions of S- and P-Oxy Radicals

Likely reactions of the neutral radicals in biological tissues are abstractions of allylic type H-atoms from unsaturated lipid components and/or additions to their C=C double bonds. Propene was chosen as a simple model of oleic acid to test the ease/difficulty of both types of reactivity for the S- and P-radicals. The Gibbs free energies were computed at the CAM-B3LYP/6-311+G(2d,p) level in vacuum. Transition states for the H-abstraction and addition (or migration, see below) reactions were located enabling free energies of activation Δ*G*^*⧧*^ to be calculated. For comparison purposes, some data were obtained for the hydroxyl (HO^•^) and bicarbonate radicals (HOCO_2_^•^) as models (see Table 4). To check on the influence of a lipid-like medium, selected examples were computed with CPCM formalism using pentanoic acid as the solvent (see Table S1 in the Supporting Information). However, using pentanoic acid as a solvent made only small differences to the computed free energies and did not affect any of the resulting conclusions.

**Table 4:** Computed Energetics for Addition and H-Atom Abstraction Reactions of S-, P-, and Model Radicals with Propene^a^

	addition C-1		allylic H-abstraction			addition C-2
Radical	Δ*G*^o^	Δ*G*^*⧧*^	Δ*G*^o^	Δ*G*^*⧧*^	*r*(C···H)^⧧,^^b^	Δ*G*^o^
^•^SO_2_OH, **4b**	2.3	11.8	9.7	23.7	1.413	5.5
HOSO_3_^•^, **7**	–12.7	3.6	–21.7	9.1	1.222	–9.2
^•^PO(OH)_2_, **10b**	–13.0	11.9	–5.0	20.5	1.353	–7.9
(HO)_2_PO_2_^•^, **13**^·^	–14.9	6.5	–24.5	13.3	1.126	–11.9
(HO)CO_2_^•^	–9.8	10.0	–20.8	5.7	1.133	
HO^•^	–19.9	4.8	–31.4	6.6	1.149	

a Free energies (kcal mol^–1^) computed at the CAM-B3LYP/6-311+G(2d,p)//CAM-B3LYP/6-311 +G(2d,p) level in vacuo.

b Length of the breaking C–H bond in the H-abstraction TS, in Å.

As a cross-check on the method, the Gibbs free energies for addition to the central atom (C-2) of propene were also computed in selected cases (Table 4). For every radical tested, the method returned Δ*G*^o^ values that were more favorable for addition at the terminal atom (C-1) (compare Table 4 column 2 with column 5). This agrees entirely with expectations^64−66^ and adds credence to the reliability of the method.

**Chart 3:**
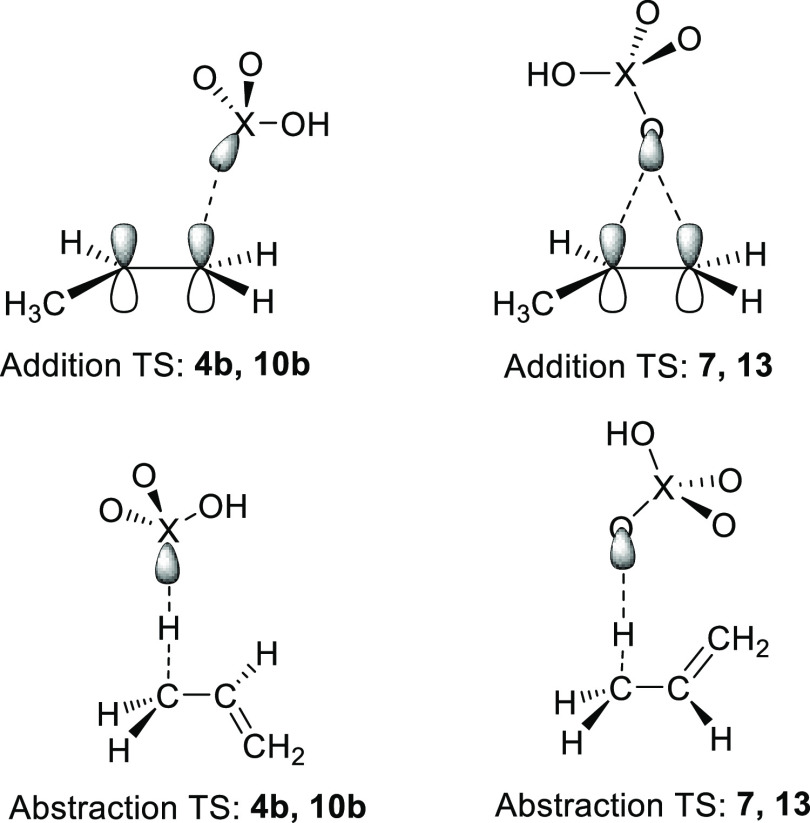
Schematic sketches of the differing TSs for additions to (or 1,2 shifts, see text) and abstractions from propene of the S- and P-radicals and the delocalized O-centered radicals (X = S or P).

The transition states for addition of S-centered radical **4b** and P-centered radical **10b** were “normal” in that the radical approached propene from “directly above” that is, perpendicular to the plane of the double bond such that overlap of the S-atom or P-atom orbital with the π-system was maximum (see structures in the Supporting Information and Chart 3). In their TSs, the computed X–C1–C2 access angles were 104.2 and 99.7°, and the S–C1 and P–C1 bond lengths were 2.411 and 2.624 Å for the two radicals, respectively. The electronic structures of radicals **7**, **13**, and HOCO_2_^•^ differ in that their upes are distributed/delocalized across [O–X=O]^•^ units. Interestingly, for all three (and for the HO^•^ radical), the TS optimizations afforded structures where the O-atom of the radical is placed “above” the plane of the propene π-system in a bridging mode (see Chart 3 and the Supporting Information). In each case, the bridge was unsymmetrical with a slightly shorter bond to propene C2. It turned out that these TSs do not describe the desired addition reactions but rather 1,2-migrations between isomeric C1- and C2-addition products. Because in all of these bridging TSs, the C···O bonds are effectively broken (e.g., for the HO^•^ radical, they were both around 2.39 Å) and the spin density is mostly located on the radical fragment (e.g., spin density of 0.78 on HO^•^), we used these results to model the actual (elusive) addition TSs.

Previous experimental research with β-(acyloxy)alkyl and β-(phosphatoxy)alkyl [^•^CR_2_CR′O–X(L_*n*_)=O, X = S, P] and related radicals demonstrated that they rapidly undergo 1,2-migrations of their [O–X=O]^•^ units.^67^ The computed migration barriers for radicals **7**, **13**, HOCO_2_^•^_,_ and HO^•^ (via the bridged TSs and relative to the more stable C1 adduct) are Δ*G*^*⧧*^ = 16.4, 21.4, 19.7, and 24.8 kcal/mol, respectively, suggesting that the propene adduct radicals, except perhaps the HO^•^ adduct radical, all have the capacity for analogous 1,2 shifts.

Transition states for H abstraction^68^ (leading to the neutral oxyacid with one more H-atom and an allyl radical) were located assuming that the atomic site with the highest spin density (O or X) accepts the H-atom. For all of radicals **4b, 10b, 7, 13,** HO^•^, and HOCO_2_^•^, the TS configurations X····H····C were close to linear, as is normal for H-abstractions (Chart 3). From the lengths of the breaking C–H bonds of propene in the TS included in Table 4, it is evident that the TSs for H-abstraction by S- and P-centered radicals **4b** and **10b** were of the “late” variety (breaking C–H bond distances 1.35–1.41 Å), whereas the TSs of the other O-centered radicals were all “early” types (breaking C–H distances 1.13–1.22 Å). This accords well with the comparatively weak S–H and P–H bonds in the H–SO_2_OH and H–PO(OH)_2_ products from the first two radicals and the comparatively strong H–O bonds in the products from the other radicals. The reaction free energies range from endergonic for S-centered radical **4b** to strongly exergonic for the HO^•^ radical. Interestingly, plots of the computed Δ*G*^*⧧*^ versus Δ*G*^o^ and Δ*H*^*⧧*^ versus Δ*H*^o^ for the series of abstractions from propene were close to linear (*R*^2^ = 0.895 and 0.837, respectively, see Figure S2 in the Supporting Information) indicating that an approximate Bell–Evans–Polanyi type relationship [Δ*H*^*⧧*^ = αΔ*H*^o^ + C]^69−72^ held for this series of reactions. The scatter is as expected for such a diverse series of radicals. The values of α (the slopes of the linear regression lines) determined from the Δ*H* and Δ*G* correlations were 0.41 and 0.44, respectively. These were only slightly less than the α-values of 0.49 and 0.53 obtained experimentally for H-abstractions from alkanes by methyl and trifluoromethyl radicals, respectively.^66,73^ These α-values, roughly halfway between zero and one, indicate that as expected, the comparatively weak C–H bond in propene was an important factor in controlling the energetics of these reactions.

**Figure 6:**
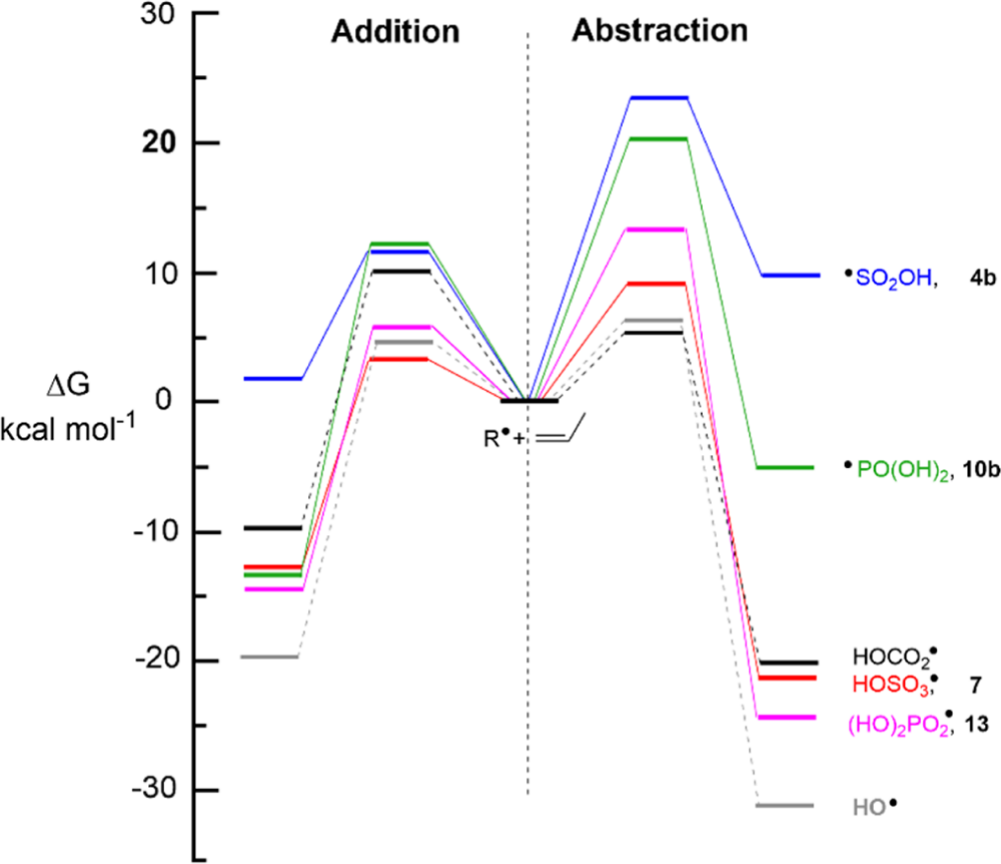
Schematic of the reaction coordinates for addition and abstraction from propene of S- and P-based radicals and model radicals (free energies from Table 4). Blue lines: ^•^SO_2_OH radicals **4b**. Green lines: ^•^PO(OH)_2_ radicals **10b**. Black lines, HCO_2_^•^ radicals. Red lines, HOSO_3_^•^ radicals **7**. Purple lines, (HO)_2_PO_2_^•^ radicals **13**. Gray lines, HO^•^ radicals.

A graphic comparison of the energetics of the two modes of the reaction is presented in Figure 6 which highlights the striking difference between the radicals having their upes mainly localized on O-atoms and those with upes localized on S- or P-atoms.

For S-centered bisulfite radical **4b**, both addition and H-abstraction are endergonic, but addition is more favorable. Reactions with lipids will be sluggish at ambient temperatures. For P-centered dihydro-phosphite radical **10b**, both addition and abstraction are exergonic, but addition will be the favored mode of reaction. The hydroxyl radical is known to be the most energetic ROS which is unselective but preferentially abstracts allylic H-atoms from unsaturated lipids.^74,75^ In agreement, Figure 6 shows HO^•^ radicals with a low activation energy and the most favorable Δ*G*^o^ for allylic abstraction. O-centered bisulfate **7**, dihydrogen phosphate, **13,** and bicarbonate HCO_2_^•^ follow this same pattern, that is, their Δ*G*^o^s for allylic abstractions are more exergonic than those of their addition reactions. It should be noted, however, that most of the computed activation energies Δ*E*^*⧧*^ on the potential energy surfaces are negative (see Table S2 and Figure S3 in the Supporting Information), suggesting that the origin of the activation free energies Δ*E*^*⧧*^ is entropic in nature and that most reactions are expected to be close to diffusion control. The interesting prediction is that in living systems, neutral bisulfate **7** and dihydro-phosphate, **13** radicals will augment the degradative activity of hydroxyl radicals with lipids. On the other hand, addition of dihydro-phosphite radicals **10a** to double bonds will damage lipids in different ways and lead to different downstream products. It is projected that the bisulfite radical **4b** will cause minimal damage to lipid components under physiological conditions. It may, of course, initiate other radical processes, particularly by deprotonation and generation of sulfite anion radicals **5**.

## Conclusions

Our ab initio and DFT computations predicted that on the one hand, the HSO_3_^•^ radical and SO_3_^–•^ anion radical would be S-centered species, whereas the HSO_4_^•^ radical and the SO_4_^–•^ anion radical would be O-centered. A similar pattern emerged for phosphorus species in that the H_2_PO_3_^•^ radical and HPO_3_^–•^ anion radical were predicted to be P-centered, but the H_2_PO_4_^•^ radical and HPO_4_^–•^ anion radical were computed to be O-centered. These predictions were upheld by experimental EPR data. From DFT computations of their Gibbs free energies of dissociation, both neutral bisulfite and bisulfate radicals ^•^SO_2_OH **4b** and HOSO_3_^•^**7** were found to be extremely strong acids, verging on the super-acid class. Corresponding P-based radicals ^•^PO(OH)_2_**10b** and (HO)_2_PO_2_^•^**13** were also found to be strong acids but more than 4 orders of magnitude less acidic than the S-based species. A study of microhydration of these species showed a distinct connection between the number of hydrating water molecules needed to induce ionization and the acidity. Extremely strong **4b** and **7** required only three and two hydrating water molecules, whereas **10b** and **13** required seven and eight water molecules to induce ionization.

Computations with propene as a simple model substrate for lipids showed that the S- and P-centered radicals would preferentially add to the double bond, whereas the O-centered species would prefer H-abstraction. The extreme acidity of both the neutral sulfur-based radicals indicated that in most biological fluids, they would deprotonate such that the principal reacting species would be the anion radicals SO_3_^–•^ (**5**) and SO_4_^–•^ (**8**). However, less acidic neutral P-radicals **10b** and **13** could well persist in lipid environments and react by addition and abstraction, respectively.

## Supporting Information Available

Computational methods, Cartesian coordinates and energies for computed structures of S- and P-based radicals and transition states, structures and energetics for microhydrated clusters, and additional graphical and tabular material (PDF)
