# Supplementary material for: Sulfur and Phosphorus Oxyacid Radicals
Source: J Phys Chem A. 2022 Jan 27;126(5):760–71. doi: 10.1021/acs.jpca.1c10455 (PMC9007452; doi:10.1021/acs.jpca.1c10455)
Supplement: Supplementary file 1 — jp1c10455_si_001.pdf [file jp1c10455_si_001.pdf]

Supporting Information For

Sulfur and Phosphorus Oxyacid Radicals.

Michael Bühl, Tallulah Hutson, Alice Missio and John C. Walton**

EaStCHEM School of Chemistry, University of St. Andrews, St. Andrews, Fife KY16 9ST, United Kingdom

TABLE OF CONTENTS

Computational Methods **S2**

Optimized Structures and Energies of P-Based Species **S2**

Optimised Structures and Energies of S-Based Species **S10**

Microhydration of S-Based Radicals **S16**

Microhydration of P-Based Radicals **S20**

Figure S1. Plot of Gibbs Free Energies for microhydration clusters of S- & P-radicals against no. of waters 'n'. **S29**

Transition States for Addition Reactions to Propene **S30**

Transition States for Abstraction Reactions from Propene **S34**

Figure S2. Plots of activation enthalpy vs. enthalpy of reaction of the H-abstraction reactions from propene and of the corresponding Gibbs free energies **S37**

Table S1. Computed energetics for addition and H-atom abstraction reactions of S-, P- and model radicals with propene showing results with pentanoic acid as solvent **S38**

Table S2. Computed potential energies for addition and H-atom abstraction reactions of S-, P- and model radicals with propene **S38**

Figure S3. Schematic of the reaction coordinates for addition and abstraction from propene of S- and P-based radicals and model radicals **S39**

References **S39**

Computational Methods

DFT calculations were carried out using the Gaussian 09 suite of programs[1].

The composite technique Gaussian-4 (G4)[2] is known for its high accuracy and was used to benchmark DFT methods. In a literature paper good results were reported for relevant species with hybrid functionals.[3] Hence a good number of exchange hybrid functionals were tested and compared with the G4 results. Geometries were optimized using the 6-31G(d,p) basis set, followed by single-point energy (SPE) calculations on the pre-optimized structures using the 6-311+G(2d,p) basis set. Twelve benchmark reactions were used to determine which DFT functional to use. These reactions included carbonic and sulfuric acid and their derivatives, as well as several radical conversions. Calculations were carried out for 23 DFT functionals and the Mean Absolute Deviation (MAD) and Root Mean Square Deviation (RMSD) were compared from each functional set with the G4 data set. The CAM-B3LYP functional[4] which combines the hybrid qualities of B3LYP with the long-range correction proposed by Tawada et al.[5] gave the best correlation results for the C-based and S-based radicals[6] in comparison with G4 (MAD: 2.5 kcal mol^{-1}). Functionals of comparable accuracy, such as BMK (MAD 3.2 kcal mol^{-1}), ωB97 (MAD 3.3 kcal mol^{-1}) and M05 (MAD 3.6 kcal mol^{-1}) displayed no significant computational time advantages.

Optimised Structures and Energies of S- and P-Based Species

$HP(=O)_2OH(.)$ Radical (10a)
File = HPO2OHg4
G4/G4 – Gas Phase
E(UHF) = -566.6434939 AU
ΔG = -568.073712 AU

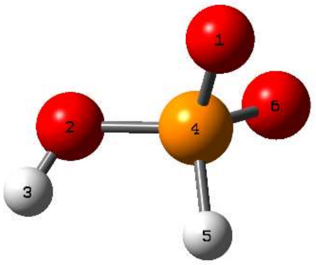

G4/G4 scrf=(CPCM, solvent=water)
File = HPO2OHg4aq
E(UHF) = -566.6602936

Center Number	Atomic Number	Atomic Type	Coordinates (Angstroms) X	Y	Z
1	8	0	0.839411	1.194167	-0.213647
2	8	0	-1.444633	-0.007284	-0.317028
3	1	0	-2.140409	0.071115	0.344971
4	15	0	0.035550	0.002303	0.259060
5	1	0	-0.166635	0.013172	1.651606
6	8	0	0.826946	-1.201737	-0.204634

$HP(OH)_2O$, 9Ha
G4/G4
File = HPOH2Og4
E(UHF) = -567.31835209
ΔG = -568.761514

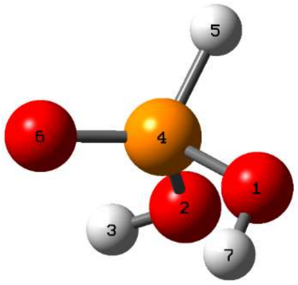

Center Number	Atomic Number	Atomic Type	Coordinates (Angstroms) X	Y	Z
1	8	0	1.439567	0.415164	-0.121266
2	8	0	-0.987617	1.092608	-0.150402
3	1	0	-1.781437	0.751992	-0.579713
4	15	0	-0.014161	-0.111108	0.269201
5	1	0	0.091796	0.045654	1.655528
6	8	0	-0.425112	-1.423106	-0.239197
7	1	0	1.687347	0.191646	-1.026909

$P(OH)_3$ 9Hb
G4/G4
File = POH3g4
E(UHF) = -567.29528742
ΔG = -568.743271

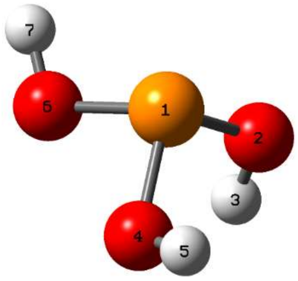

Center Number	Atomic Number	Atomic Type	Coordinates (Angstroms) X	Y	Z
1	15	0	0.000055	-0.010535	0.507289
2	8	0	-0.014821	-1.486643	-0.184856
3	1	0	-0.014759	-1.445426	-1.152710
4	8	0	-1.178604	0.768437	-0.328969
5	1	0	-2.014808	0.713111	0.144037
6	8	0	1.193527	0.744945	-0.329387

7	1	0	2.027932	0.676427	0.145039

$OP(OH)_2(-)$ Anion, 9b
G4/G4 Vacuum
File+ OPOH2ag4
E(UHF) = -566.72736602
ΔG = -568.203827

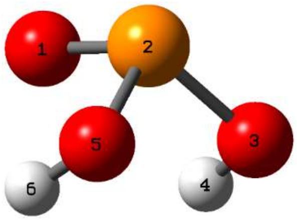

Center Number	Atomic Number	Atomic Type	Coordinates (Angstroms) X	Y	Z
1	8	0	0.000273	-1.390454	-0.331336
2	15	0	-0.000137	-0.114953	0.503060
3	8	0	-1.303734	0.763193	-0.177443
4	1	0	-1.431552	0.317153	-1.028905
5	8	0	1.303634	0.763617	-0.177341
6	1	0	1.432220	0.316300	-1.028039

(.)$PO(OH)_2$(.) radical (10b)
G4/G4 Gas Phase
File = OPOH2g4
E(UHF) = -566.68461276
ΔG = -568.113738

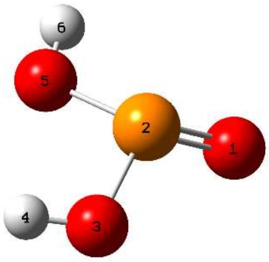

G4/G4 scrf=(CPCM, solvent=water)
File = OPOH2g4aq
E(UHF) = -566.7018479

Center Number	Atomic Number	Atomic Type	Coordinates (Angstroms) X	Y	Z
1	8	0	1.499173	-0.307071	-0.240542
2	15	0	0.153435	-0.048616	0.308007

3	8	0	-0.974959	-1.102934	-0.153960
4	1	0	-1.861668	-0.720608	-0.153909
5	8	0	-0.582135	1.330938	-0.148490
6	1	0	0.023500	2.082397	-0.122269

$HPO_2OH(-)$ Anion (9a)
G4/G4
File = HPO2OHag4
E(UHF) = -566.77041137
ΔG = -568.237979

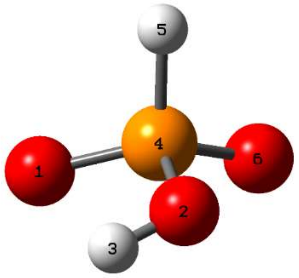

Center Number	Atomic Number	Atomic Type	Coordinates (Angstroms) X	Y	Z
1	8	0	0.515885	-1.374449	-0.200258
2	8	0	0.992197	1.102703	-0.096519
3	1	0	1.742005	0.610088	-0.445964
4	15	0	-0.131242	-0.091450	0.214542
5	1	0	-0.122416	-0.017918	1.646053
6	8	0	-1.464451	0.369193	-0.255500

$HOPO_2(-)(.)$ Anion Radical, 11a
G4/G4 vacuum
File: HOPO2arg4
E(UHF) = -566.14526086
ΔG = -567.598576

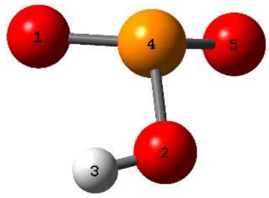

Center Number	Atomic Number	Atomic Type	Coordinates (Angstroms) X	Y	Z
1	8	0	-0.564444	1.365796	-0.178667
2	8	0	-0.958411	-1.144889	-0.081252
3	1	0	-1.760222	-0.661661	-0.312920
4	15	0	0.135712	0.104972	0.273095
5	8	0	1.488423	-0.335022	-0.213019

HPO_3(-)(.) Anion Radical 11b
G4/G4
File: HPO3arg4
E(UHF) = 566.1052061
ΔG = -567.573818

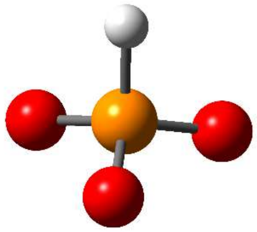

Center Number	Atomic Number	Atomic Type	Coordinates (Angstroms) X	Y	Z
1	8	0	1.332571	-0.593713	-0.206563
2	8	0	-0.151629	1.450661	-0.206214
3	15	0	0.000043	-0.000316	0.220304
4	8	0	-1.180975	-0.856179	-0.206651
5	1	0	-0.000367	-0.001415	1.650866

$(OH)_2PO_2$(.) Radical (13)
G4/G4 vacuum
File: HO2pO2g4b
E(UHF) = -641.59946595
ΔG = -643.335434

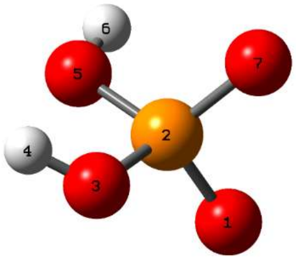

G4/G4 – CPCM(water)
File = HO2pO2g4aq
E(UHF) = -641.61531645
ΔG = -643.348690

Center Number	Atomic Number	Atomic Type	Coordinates (Angstroms) X	Y	Z
1	8	0	1.025397	-0.248901	-1.153358
2	15	0	0.077575	-0.026369	0.002528
3	8	0	-1.012626	-1.165717	0.095375
4	1	0	-1.908890	-0.841233	0.236092
5	8	0	-0.871133	1.245626	-0.154036
6	1	0	-0.386949	2.079861	-0.134837
7	8	0	0.999888	0.063607	1.194622

$(HO)_3PO$, (12H)
G4/G4
File = HO3POg4
E(UHF) = -642.26740678
ΔG = -644.016334

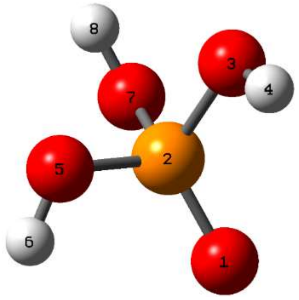

Center Number	Atomic Number	Atomic Type	Coordinates (Angstroms) X	Y	Z
1	8	0	-1.030039	-0.106737	1.218053
2	15	0	-0.078633	-0.015619	0.116681
3	8	0	0.876541	1.258980	0.035577
4	1	0	0.399349	2.086775	0.161156
5	8	0	-0.742881	-0.001144	-1.344122
6	1	0	-1.607202	-0.428253	-1.326806
7	8	0	0.991383	-1.188909	0.095628
8	1	0	1.627320	-1.121773	-0.625653

$(HO)_2PO_2(-)$ anion, (12)
G4/G4 vacuum
File = HO2pO2ag4
E(UHF) = -641.72052786
ΔG = -643.494489

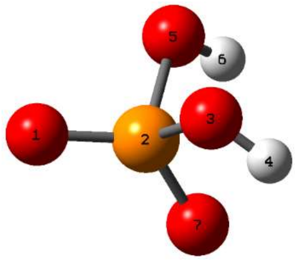

Center Number	Atomic Number	Atomic Type	Coordinates (Angstroms) X	Y	Z
1	8	0	0.003305	1.580843	0.270515
2	15	0	0.000223	0.107240	0.134681
3	8	0	1.281170	-0.313166	-0.835413

4	1	0	1.588558	-1.145695	-0.464825
5	8	0	-1.282380	-0.307973	-0.835608
6	1	0	-1.593374	-1.138907	-0.464452
7	8	0	-0.001912	-0.875204	1.264140

$HOPO_3$(-)(.) Anion radical (14)
G4/G4 vacuum
File = HOPO3arg4
E(UHF) = -641.06963190
ΔG = -642.830768

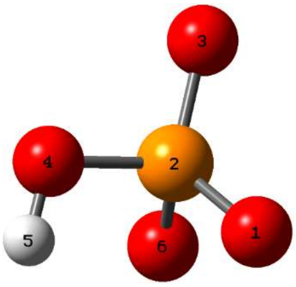

Center Number	Atomic Number	Atomic Type	Coordinates (Angstroms) X	Y	Z
1	8	0	0.891363	-0.377516	1.184225
2	15	0	0.047544	0.117488	0.000502
3	8	0	-0.405913	1.532929	0.002281
4	8	0	-1.334750	-0.789369	-0.002229
5	1	0	-1.061425	-1.711768	-0.002187
6	8	0	0.892834	-0.372363	-1.184945

$HOPO_2$(-)(.) Anion Radical (11)
G4/G4
File = HOPO2arg4
E(UHF) = -566.14526086
ΔG = -567.598576

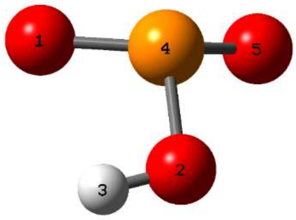

Center Number	Atomic Number	Atomic Type	Coordinates (Angstroms) X	Y	Z
1	8	0	-0.564444	1.365796	-0.178667
2	8	0	-0.958411	-1.144889	-0.081252
3	1	0	-1.760222	-0.661661	-0.312920

4	15	0	0.135712	0.104972	0.273095
5	8	0	1.488423	-0.335022	-0.213019

Optimised Structures and Energies of S-Based Species

HSO_3(.) Radical (4a)
G4/G4 vacuum
File: HSO3g4
E(UHF) = -622.71048435
ΔG = -624.207864

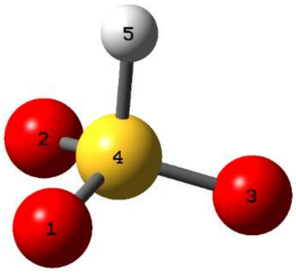

G4/G4 CPCM, water
E(UHF) = -622.71898105
ΔG = -624.215020

Center Number	Atomic Number	Atomic Type	Coordinates (Angstroms) X	Y	Z
1	8	0	1.173598	-0.762895	-0.206660
2	8	0	-1.248045	-0.633858	-0.206679
3	8	0	0.074591	1.397594	-0.206384
4	16	0	-0.000078	-0.000403	0.211934
5	1	0	0.000097	-0.000274	1.566843

HSO_3(-) Anion (3a)
G4/G4 vacuum
File: HSO3ag4
E(UHF) = -622.86925230
ΔG = -624.380838

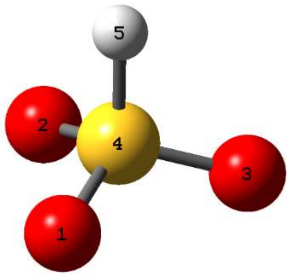

Center Number	Atomic Number	Atomic Type	Coordinates (Angstroms) X	Y	Z

1	8	0	-0.755366	-1.205213	-0.175967
2	8	0	-0.666436	1.256555	-0.175930
3	8	0	1.421668	-0.051323	-0.175921
4	16	0	0.000141	-0.000010	0.167028
5	1	0	-0.001183	0.000014	1.550097

(.)SO_2OH Radical (4b)
G4/G4 vacuum
File: SO2OHg4
E(UHF) = -622.76821250
ΔG = -624.252356

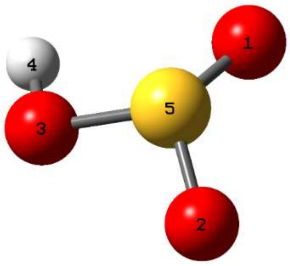

G4/G4 CPCM, water
E(UHF) = -622.78038864
ΔG = -624.261206

Center Number	Atomic Number	Atomic Type	Coordinates (Angstroms) X	Y	Z
1	8	0	0.205546	-1.421760	-0.188147
2	8	0	-1.310687	0.631429	-0.198625
3	8	0	1.128318	0.900698	-0.107551
4	1	0	1.924172	0.346367	-0.136710
5	16	0	-0.131849	-0.076832	0.255706

SO_3(-.) Radical Anion (5)
G4/G4 vacuum
File: SO3g4ra
E(UHF) = -622.26026130
ΔG = -623.759173

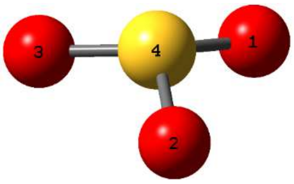

Center Number	Atomic Number	Atomic Type	Coordinates (Angstroms) X	Y	Z

1	8	0	0.991672	1.042990	-0.150630
2	8	0	0.407636	-1.380157	-0.150630
3	8	0	-1.399163	0.337132	-0.150669
4	16	0	-0.000072	0.000018	0.225964

$(HO)_2SO$ (3Hb)
G4/G4 vacuum
File: SOOH2g4
E(UHF) = -623.39133217
ΔG = -624.889599

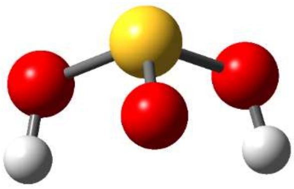

Center Number	Atomic Number	Atomic Type	Coordinates (Angstroms) X	Y	Z
1	8	0	0.003646	-1.369172	-0.312647
2	8	0	1.258794	0.752233	-0.149430
3	1	0	1.471330	0.404059	-1.032117
4	16	0	0.000017	-0.115144	0.434921
5	8	0	-1.262299	0.746706	-0.149578
6	1	0	-1.472731	0.400109	-1.033387

HSO_2OH (3Ha)
G4/G4 vacuum
File = HSO2OHg4
E(UHF) = -623.38386995
ΔG = -624.878514

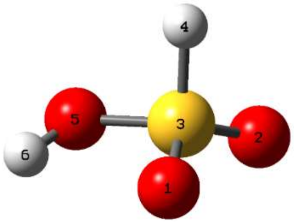

Center Number	Atomic Number	Atomic Type	Coordinates (Angstroms) X	Y	Z
1	8	0	0.257533	1.393839	-0.201620
2	8	0	-1.319708	-0.572577	-0.245346
3	16	0	-0.127245	0.071068	0.200287
4	1	0	-0.109853	0.013189	1.555903
5	8	0	1.099238	-0.917702	-0.099120

6	1	0	1.849264	-0.378761	-0.391807

$HOSO_3$(.) Radical (7)
G4/G4 vacuum
File = HOSO3g4
E = -697.64346122
ΔG = -699.434678

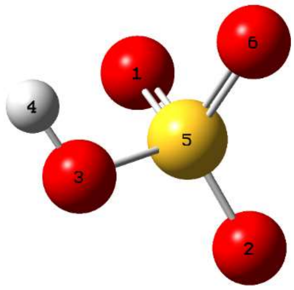

Center Number	Atomic Number	Atomic Type	Coordinates (Angstroms) X	Y	Z
1	8	0	-0.827750	-0.448299	-1.135577
2	8	0	0.229544	1.496918	0.000613
3	8	0	1.371028	-0.605336	-0.000330
4	1	0	1.257999	-1.568268	-0.000749
5	16	0	-0.051158	0.101206	0.000180
6	8	0	-0.827756	-0.449662	1.135029

$HOSO_3$(-) Anion (6)
G4/G4 vacuum
File = HOSO3ag4
E = -697.79031531
ΔG = -699.611632

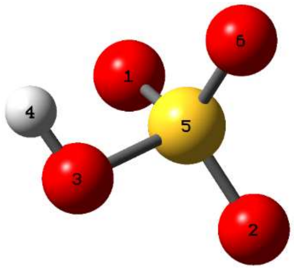

Center Number	Atomic Number	Atomic Type	Coordinates (Angstroms) X	Y	Z
1	8	0	0.281537	-0.811268	1.224190
2	8	0	0.835746	1.256248	-0.000204
3	8	0	-1.457099	0.463090	0.000152

4	1	0	-1.949846	-0.365751	0.000125
5	16	0	0.151172	-0.025443	-0.000014
6	8	0	0.281203	-0.811466	-1.224126

SO4(-.) Anion Radical (8)
G4/G4
File = SO4rag4
E = -697.14575466
ΔG = -698.951587

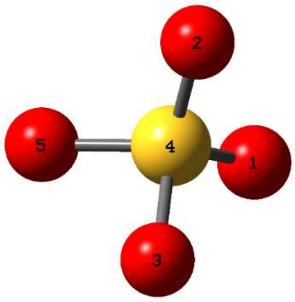

Center Number	Atomic Number	Atomic Type	Coordinates (Angstroms) X	Y	Z
1	8	0	0.869801	-1.224393	-0.003737
2	8	0	0.871999	1.222722	0.001969
3	8	0	-0.949087	-0.002465	1.117662
4	16	0	0.079281	0.000175	0.000374
5	8	0	-0.951275	0.003786	-1.116641

$HOSO_2$(-) anion (3b)
G4/G4
File = HOSO2ag4
E = -622.84892927
ΔG = -624.373833

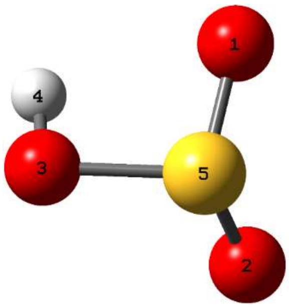

Center Number	Atomic Number	Atomic Type	Coordinates (Angstroms) X	Y	Z
1	8	0	0.062062	1.384852	-0.256664

2	8	0	1.145742	-0.841266	-0.334526
3	8	0	-1.387549	-0.619300	-0.068613
4	1	0	-1.602879	-0.018866	-0.796946
5	16	0	0.190052	0.039037	0.379711

$(HO)_2SO_2$ Acid (6H)
G4/G4 vacuum
File = OH2SO2bg4
E(UHF) = -698.30271305
ΔG = -700.106204

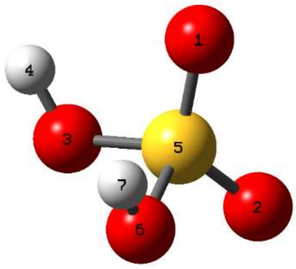

Center Number	Atomic Number	Atomic Type	Coordinates (Angstroms) X	Y	Z
1	8	0	-0.002450	-0.837675	1.230459
2	8	0	0.002992	1.497471	0.271913
3	8	0	-1.239855	-0.269734	-0.813469
4	1	0	-1.575937	-1.140039	-0.555504
5	16	0	0.000122	0.085192	0.131026
6	8	0	1.239769	-0.274510	-0.812038
7	1	0	1.570332	-1.147449	-0.555831

Microhydration of S-Based Radicals

CAM-B3LYP/6-311+G(2d,p) with CPCM(water)

SO_2OH(.) 1xH_2O

File asof

E(UHF) = -700.88847910

ΔG =-700.874334

Center Number	Atomic Number	Atomic Type	Coordinates (Angstroms) X	Y	Z
1	16	0	-0.773779	0.057175	-0.248365
2	8	0	0.363689	-0.936328	0.208327
3	1	0	1.296151	-0.527442	0.093812
4	8	0	-0.455836	1.412388	0.172026
5	8	0	-2.031025	-0.550141	0.143915
6	8	0	2.704500	0.039703	-0.081980
7	1	0	3.372307	-0.603527	-0.351704
8	1	0	3.061392	0.491198	0.693426

SO_2OH(.) 2xH_2O

File 2cso

E(UHF) = -777.34137720

ΔG = -777.306261

Center Number	Atomic Number	Atomic Type	Coordinates (Angstroms) X	Y	Z
1	16	0	-1.161973	0.119233	0.251607
2	8	0	-0.557636	-1.234579	-0.234472
3	1	0	0.500969	-1.302988	-0.109553
4	8	0	-2.550594	0.157463	-0.165693
5	8	0	-0.289191	1.235072	-0.111177
6	8	0	1.900090	-1.427896	-0.034164
7	1	0	2.206056	-1.918001	0.738667
8	1	0	2.305845	-0.521661	0.003799
9	1	0	1.690622	1.466976	-0.040040
10	8	0	2.595160	1.127105	0.052015
11	1	0	3.105445	1.510629	-0.670656

SO_2OH(.) 3xH_2O

File 3aso

E(UHF) = -853.79547643

ΔG = -853.735265

Center Number	Atomic Number	Atomic Type	Coordinates (Angstroms) X	Y	Z
1	16	0	1.428526	-0.087155	0.108897

2	8	0	0.739864	-0.063186	1.432160
3	1	0	-0.893993	0.034110	1.321251
4	8	0	1.280611	1.183877	-0.633590
5	8	0	1.091185	-1.287066	-0.688351
6	8	0	-1.811621	0.099669	0.914798
7	1	0	-1.875831	-0.731519	0.302804
8	1	0	-1.764714	0.944199	0.321322
9	1	0	-1.808344	2.285201	-1.321373
10	8	0	-1.349430	2.154148	-0.482669
11	1	0	-0.405922	2.000288	-0.692772
12	1	0	-1.900628	-2.762304	-0.285973
13	8	0	-1.642573	-1.895535	-0.622843
14	1	0	-0.671274	-1.910745	-0.743641

$SO_2OH(.)$ 4xH_2O

File 4asob & 4asod

E(UHF) = -930.24543708

ΔG = -930.167319

Center Number	Atomic Number	Atomic Type	Coordinates (Angstroms) X	Y	Z
1	16	0	1.716851	-0.776322	-0.226352
2	8	0	1.774716	-0.247854	1.165733
3	1	0	0.443429	0.704283	1.588739
4	8	0	1.816688	0.291429	-1.247476
5	8	0	0.583334	-1.702053	-0.443003
6	8	0	-0.432528	1.170794	1.479344
7	1	0	-1.110052	0.386159	1.183706
8	1	0	-0.283954	1.789231	0.680372
9	1	0	-0.419651	2.766031	-1.246720
10	8	0	0.242351	2.513457	-0.592080
11	1	0	0.821074	1.846777	-1.015056
12	1	0	-2.591509	-0.606574	0.223525
13	8	0	-1.788443	-0.755123	0.781199
14	1	0	-1.139938	-1.263002	0.257369
15	8	0	-4.028212	-0.301670	-0.619697
16	1	0	-4.599422	-1.066834	-0.757083
17	1	0	-3.932839	0.113242	-1.485372

$SO_3(.)OH$ 1x H_2O

File= 1so4b

E(UHF) = -776.08142543

ΔG = -776.065622

Center Number	Atomic Number	Atomic Type	Coordinates (Angstroms) X	Y	Z
1	16	0	0.592723	0.072577	-0.004235
2	8	0	-0.487458	-1.011680	0.047685

3	1	0	-1.453224	-0.615805	0.057614
4	8	0	-0.011845	1.366970	-0.042235
5	8	0	1.517906	-0.258987	-1.115617
6	8	0	-2.795605	-0.048001	0.084936
7	1	0	-3.407494	-0.413391	-0.567415
8	1	0	-2.783679	0.908377	-0.054607
9	8	0	1.547105	-0.178353	1.104252

SO_3(.)OH 2x H_2O
File= 2so4b
E(UHF) = -852.53809148
DG = -852.499823

Center Number	Atomic Number	Atomic Type	Coordinates (Angstroms) X	Y	Z
1	16	0	0.981190	-0.036380	0.008738
2	8	0	0.248028	1.237796	-0.050739
3	1	0	-1.217751	1.307913	-0.045712
4	8	0	0.101659	-1.184083	0.129264
5	8	0	1.940555	-0.157162	-1.135216
6	8	0	-2.254647	1.281721	-0.078580
7	1	0	-2.621453	1.757689	0.681855
8	1	0	-2.534476	0.252760	-0.048210
9	1	0	-3.150933	-1.554485	0.601652
10	8	0	-2.631073	-1.148205	-0.103569
11	1	0	-1.698343	-1.421202	0.021779
12	8	0	2.035969	-0.000141	1.069944

SO_3(.)OH 3x H_2O
File= 3so4f
E(UHF) = -928.99063700
ΔG = -928.928638

Center Number	Atomic Number	Atomic Type	Coordinates (Angstroms) X	Y	Z
1	16	0	1.190132	-0.018572	0.034630
2	8	0	0.528674	0.038578	1.341204
3	1	0	-1.081673	-0.041139	1.285791
4	8	0	0.883163	1.193148	-0.793970
5	8	0	0.921624	-1.255307	-0.677698
6	8	0	-2.037320	-0.092452	0.968700
7	1	0	-2.054971	-0.925954	0.353408
8	1	0	-2.172021	0.749262	0.403433
9	1	0	-2.690904	2.151791	-1.146508
10	8	0	-2.086889	2.063593	-0.398368
11	1	0	-1.192279	2.179381	-0.751918
12	1	0	-1.949164	-2.948344	-0.225677

13	8	0	-1.760149	-2.066949	-0.570277
14	1	0	-0.792797	-2.010201	-0.704537
15	8	0	2.662359	0.262183	0.159400

SO_3(.)OH 4x H_2O

File= 4so4a

E(UHF) = -1005.43772407

ΔG = -1005.363252

Center Number	Atomic Number	Atomic Type	Coordinates (Angstroms) X	Y	Z
1	16	0	0.938255	-0.492690	-0.402672
2	8	0	0.230989	0.724065	-0.001594
3	1	0	-1.255160	0.751243	0.499827
4	8	0	1.452875	-0.375451	-1.817912
5	8	0	0.163930	-1.703212	-0.208373
6	8	0	-2.196647	0.662097	0.856662
7	1	0	-2.368852	-0.351511	0.954283
8	1	0	-2.868028	1.096751	0.225765
9	1	0	-4.640915	2.180223	-0.266250
10	8	0	-3.857065	1.819932	-0.699896
11	1	0	-4.175784	1.308497	-1.454350
12	1	0	-2.390819	-2.263140	1.830762
13	8	0	-2.336607	-1.847029	0.961901
14	1	0	-1.439357	-2.021914	0.613238
15	8	0	2.297769	-0.568010	0.216788
16	8	0	3.765529	1.840968	1.015598
17	1	0	4.675618	1.703138	0.733465
18	1	0	3.285036	1.052880	0.720618

Microhydration of P-Based Radicals
CAM-B3LYP/6-311+G(2d,p) with CPCM(water)

$(HO)_2PO_2$(.) $1H_2O$
File = H2PO4aq1b
E(UHF) = -720.00386573
DG = -719.978283

Center Number	Atomic Number	Atomic Type	Coordinates (Angstroms) X	Y	Z
1	15	0	-0.609519	-0.031165	0.000575
2	8	0	-0.641244	-1.027210	-1.144305
3	8	0	-1.802700	0.995287	-0.014601
4	1	0	-2.678281	0.584002	-0.010389
5	8	0	0.575232	0.961937	-0.006441
6	1	0	1.504123	0.562800	-0.002851
7	8	0	-0.650300	-1.002181	1.166371
8	8	0	2.939290	-0.052281	0.009211
9	1	0	3.444365	0.068297	-0.804562
10	1	0	3.510354	0.247957	0.727305

$(HO)_2PO_2$(.) $2H_2O$
File = h2po4aq2b
E(UHF) = -796.45758885
DG = -796.413001

Center Number	Atomic Number	Atomic Type	Coordinates (Angstroms) X	Y	Z
1	15	0	-0.000064	-0.015862	0.007141
2	8	0	0.000548	-0.983341	1.181577
3	8	0	1.195095	0.977050	-0.015225
4	1	0	2.111746	0.566252	-0.011648
5	8	0	-1.194958	0.977419	-0.013913
6	1	0	-2.111675	0.566744	-0.010808
7	8	0	-0.000963	-1.034255	-1.123119
8	8	0	-3.575825	-0.061712	-0.005850
9	1	0	-4.151319	0.238012	0.708651
10	1	0	-4.076833	0.060748	-0.821631
11	8	0	3.576181	-0.061851	-0.005814
12	1	0	4.077418	0.060295	-0.821510
13	1	0	4.151011	0.239414	0.708582

$(HO)_2PO_2$(.)$3H_2O$
File = h2po4aq3a
E(UHF) = -872.91142256
ΔG = -872.843221

Center Number	Atomic Number	Atomic Type	Coordinates (Angstroms) X	Y	Z
1	15	0	0.383330	-0.299752	0.067418
2	8	0	0.481114	-0.026037	1.581511

3	8	0	-0.589151	-1.445623	-0.297439
4	1	0	-1.585599	-1.262962	-0.154232
5	8	0	1.715484	-0.814883	-0.537273
6	1	0	2.530169	-0.247093	-0.374290
7	8	0	-0.044296	1.083740	-0.369799
8	8	0	-3.064091	-0.931686	0.044047
9	1	0	-3.137131	0.049535	-0.054689
10	1	0	-3.646988	-1.326809	-0.614557
11	1	0	-3.022362	2.306635	0.483281
12	8	0	-2.777601	1.719887	-0.240908
13	1	0	-1.801991	1.687855	-0.253251
14	1	0	4.136449	0.747877	0.719498
15	8	0	3.885055	0.557285	-0.192763
16	1	0	3.925396	1.399781	-0.662039

$(HO)_2PO_2(.)4H_2O$

File = h2po4aq4a

E(UHF) = -949.365394

ΔG = -949.273549

Center Number	Atomic Number	Atomic Type	Coordinates (Angstroms) X	Y	Z
1	15	0	0.001558	-0.706806	0.052874
2	8	0	-0.002565	0.791135	-0.148592
3	8	0	1.204694	-1.467043	-0.546735
4	1	0	2.133528	-1.071475	-0.363712
5	8	0	-1.206397	-1.471098	-0.531307
6	1	0	-2.134431	-1.075775	-0.341812
7	8	0	0.012191	-0.716912	1.609486
8	8	0	3.483813	-0.404867	-0.189708
9	1	0	3.292490	0.561158	-0.099032
10	1	0	3.966664	-0.674574	0.600150
11	1	0	1.569338	1.813979	-0.082053
12	8	0	2.503267	2.091186	-0.133990
13	1	0	2.653292	2.683798	0.610987
14	8	0	-3.482214	-0.409837	-0.162627
15	1	0	-3.292609	0.557667	-0.082505
16	1	0	-3.967409	-0.672228	0.628204
17	8	0	-2.510872	2.083402	-0.003041
18	1	0	-2.682086	2.699561	-0.724034
19	1	0	-1.577495	1.812242	-0.087198

$(HO)_2PO_2(.)5H_2O$

File = h2po4aq6b

E(UHF) = -1025.81445404

ΔG = -1025.701388

Center Number	Atomic Number	Atomic Type	Coordinates (Angstroms) X	Y	Z
1	15	0	-0.296387	-0.803337	0.155347
2	8	0	-0.267749	0.345817	-0.847069
3	8	0	0.703189	-1.941433	-0.126151
4	1	0	1.670833	-1.673711	-0.367507
5	8	0	-1.643565	-1.546965	0.270191
6	1	0	-2.483376	-0.963071	0.366284
7	8	0	0.041494	0.046587	1.394905
8	8	0	3.034281	-1.263841	-0.814692
9	1	0	2.997037	-0.276198	-0.924788
10	1	0	3.737091	-1.454860	-0.182768
11	1	0	1.609577	1.262286	-1.288224
12	8	0	2.547819	1.346674	-1.060253
13	1	0	2.539899	1.662225	-0.130428
14	8	0	-3.717709	-0.093797	0.371730
15	1	0	-3.474321	0.724717	-0.126984
16	1	0	-4.020709	0.182780	1.244285

17	8	0	-2.637224	1.919576	-1.032652
18	1	0	-2.946702	2.089197	-1.929543
19	1	0	-1.756903	1.511479	-1.118140
20	8	0	2.206080	1.935820	1.611315
21	1	0	2.915716	1.739405	2.233095
22	1	0	1.524734	1.258292	1.755929

$(HO)_2PO_2(.)6H_2O$
File = h2po4aq6b
E(UHF) = -1102.26697024
ΔG = -1102.136949

Center Number	Atomic Number	Atomic Type	Coordinates (Angstroms) X	Y	Z
1	15	0	0.036902	-0.951677	-0.053513
2	8	0	0.222174	-0.935297	1.495319
3	8	0	-1.223279	-1.728643	-0.484556
4	1	0	-2.123240	-1.511575	-0.029520
5	8	0	1.162788	-1.721161	-0.770687
6	1	0	2.147217	-1.456894	-0.610691
7	8	0	0.002353	0.539907	-0.282706
8	8	0	-3.444537	-1.296637	0.619929
9	1	0	-3.845600	-0.404058	0.437498
10	1	0	-4.099525	-1.958643	0.371151
11	1	0	-4.735172	1.615245	0.825968
12	8	0	-4.372076	1.123356	0.081527
13	1	0	-3.582902	1.626821	-0.231202
14	8	0	3.605019	-1.231961	-0.425369
15	1	0	3.873659	-0.332774	-0.092710
16	1	0	4.117382	-1.400695	-1.224187
17	8	0	4.251652	1.178502	0.453024
18	1	0	3.428007	1.722306	0.442695
19	1	0	4.594550	1.213845	1.352547
20	8	0	-2.103488	2.312926	-0.760546
21	1	0	-2.068417	2.556485	-1.692111
22	1	0	-1.375906	1.676927	-0.618209
23	8	0	1.904725	2.496714	0.336643
24	1	0	1.839688	3.196684	-0.322250
25	1	0	1.234080	1.829828	0.093084

$(HO)_2PO_2(.)7H_2O$
File = h2po4aq7b
E(UHF) = -1178.71484943
ΔG = -1178.558503

Center Number	Atomic Number	Atomic Type	Coordinates (Angstroms) X	Y	Z
1	15	0	0.228913	-0.437796	-0.669169
2	8	0	0.843664	0.975034	-0.772196
3	8	0	1.228958	-1.530093	-1.161482
4	1	0	2.159784	-1.479414	-0.748165

5	8	0	-1.072070	-0.656719	-1.375890
6	1	0	-2.305564	-0.163822	-0.886976
7	8	0	0.139526	-0.407106	0.883225
8	8	0	3.546011	-1.336703	-0.063306
9	1	0	3.794913	-0.408708	-0.263803
10	1	0	3.323392	-1.351245	0.892354
11	1	0	4.032450	1.665900	-1.477060
12	8	0	3.652906	1.340040	-0.653683
13	1	0	2.685222	1.355407	-0.773385
14	8	0	2.410780	-1.166309	2.433350
15	1	0	1.544950	-0.936448	2.050264
16	1	0	2.264283	-1.941643	2.986172
17	8	0	-3.178173	0.250997	-0.460378
18	1	0	-3.844941	-0.463785	-0.224877
19	1	0	-2.854855	0.744760	0.383071
20	1	0	-1.390261	0.782068	1.706648
21	8	0	-2.101832	1.416249	1.519141
22	1	0	-1.631619	2.188332	1.120430
23	1	0	-4.608689	-2.405621	0.444506
24	8	0	-4.948947	-1.549722	0.155904
25	1	0	-5.598944	-1.739249	-0.532003
26	1	0	-0.036570	2.535011	-0.256568
27	8	0	-0.633171	3.206512	0.126031
28	1	0	-0.068455	3.847972	0.571200

$(HO)_2PO_2(.)8H_2O$

File = h2po4aq8b

E(UHF) = -1255.16170085

ΔG = -1254.988151

Center Number	Atomic Number	Atomic Type	Coordinates (Angstroms) X	Y	Z
1	15	0	-0.135393	-0.297627	-0.596175
2	8	0	0.178123	1.196879	-0.822320
3	8	0	1.090921	-1.195141	-0.970031
4	1	0	1.964238	-0.913630	-0.554675
5	8	0	-1.330885	-0.850952	-1.302509
6	1	0	-2.675199	-0.587317	-0.890101
7	8	0	-0.278456	-0.152331	0.944183
8	8	0	3.303719	-0.380316	0.171415
9	1	0	3.327500	0.560000	-0.122730
10	1	0	3.025638	-0.360310	1.116395
11	1	0	3.192738	2.478079	-1.510152
12	8	0	2.840330	2.157187	-0.672688
13	1	0	1.895444	1.970580	-0.825322
14	8	0	2.051533	-0.245495	2.579864
15	1	0	1.163754	-0.252866	2.178237
16	1	0	2.071754	-0.976307	3.207552
17	8	0	-3.622786	-0.323694	-0.521587
18	1	0	-4.146754	-1.135384	-0.239755
19	1	0	-3.432666	0.293492	0.282467
20	1	0	-2.047960	0.741619	1.614924
21	8	0	-2.871080	1.192047	1.362432
22	1	0	-2.562632	2.012560	0.906660
23	1	0	-4.562366	-3.114957	0.594991
24	8	0	-5.040291	-2.374142	0.201988
25	1	0	-5.593716	-2.751868	-0.492796
26	1	0	-1.029879	2.588795	-0.469661
27	8	0	-1.772099	3.139700	-0.157147
28	1	0	-1.378224	3.925064	0.238672
29	8	0	5.650283	-1.826476	-0.329721
30	1	0	6.367630	-1.417085	0.164027
31	1	0	4.857088	-1.294188	-0.127137

$(HO)_2PO(.)\ 1H_2O$

File = oh2po1a

E(UHF) = -644.76400610

ΔG = -644.740497

Center Number	Atomic Number	Atomic Type	Coordinates (Angstroms) X	Y	Z
1	15	0	-0.703061	0.116384	-0.293907
2	8	0	-0.723220	1.512186	0.206724
3	8	0	-1.953455	-0.798120	0.137872
4	1	0	-2.803369	-0.361643	-0.014491
5	8	0	0.441593	-0.840146	0.227502
6	1	0	1.374025	-0.488048	0.116529
7	8	0	2.880540	0.048198	-0.060091
8	1	0	3.340559	0.264328	0.760363
9	1	0	3.471032	-0.537336	-0.549856

$(HO)_2PO(.)\ 2H_2O$

File = oh2po2b

E(UHF) = -721.21602333

ΔG = -721.174511

Center Number	Atomic Number	Atomic Type	Coordinates (Angstroms) X	Y	Z
1	15	0	-0.002013	0.051890	-0.237685
2	8	0	-0.020819	1.453111	0.257848
3	8	0	-1.198451	-0.882694	0.226043
4	1	0	-2.106392	-0.491158	0.090155
5	8	0	1.209341	-0.857370	0.237475
6	1	0	2.111670	-0.456925	0.089174
7	8	0	3.607743	0.158917	-0.153501
8	1	0	4.082481	0.420684	0.644824
9	1	0	4.211110	-0.411482	-0.645170
10	8	0	-3.653014	0.023143	-0.074185
11	1	0	-3.877807	0.823045	0.416502
12	1	0	-3.949270	0.176630	-0.979662

$(HO)_2PO(.)\ 3H_2O$

File = oh2po3a

E(UHF) = -797.67201486

ΔG = -797.605826

Center Number	Atomic Number	Atomic Type	Coordinates (Angstroms) X	Y	Z
1	15	0	0.417742	-0.326767	0.303077
2	8	0	-0.561364	-1.496365	-0.100566
3	1	0	-1.547909	-1.273852	-0.017435
4	8	0	1.773666	-0.877487	-0.298255
5	1	0	2.566293	-0.281220	-0.163922
6	8	0	0.017799	1.045681	-0.134574
7	8	0	-3.059844	-0.866829	0.101730
8	1	0	-3.075311	0.117717	0.009639
9	1	0	-3.623670	-1.223430	-0.594009
10	1	0	-2.857771	2.352091	0.591387
11	8	0	-2.620618	1.767757	-0.136836
12	1	0	-1.646060	1.652639	-0.097772
13	1	0	4.201794	0.789274	0.887030
14	8	0	3.924548	0.593457	-0.016354

15	1	0	3.922997	1.438571	-0.482237

(HO)$_2$PO(.) 4H_2O
File = oh2po4a
E(UHF) = -874.12742133
ΔG = -874.037875

Center Number	Atomic Number	Atomic Type	Coordinates (Angstroms) X	Y	Z
1	15	0	0.003625	-0.786232	0.320144
2	8	0	-0.001147	0.683598	-0.000375
3	8	0	1.214585	-1.602845	-0.266086
4	1	0	2.126169	-1.164001	-0.152171
5	8	0	-1.220178	-1.604222	-0.235650
6	1	0	-2.129270	-1.163193	-0.108318
7	8	0	3.488778	-0.421877	-0.077572
8	1	0	3.261021	0.538834	-0.025262
9	1	0	4.021099	-0.628636	0.699062
10	1	0	1.495638	1.695611	-0.013334
11	8	0	2.410808	2.035193	-0.096103
12	1	0	2.541058	2.650636	0.633635
13	8	0	-3.488423	-0.420586	-0.022900
14	1	0	-3.260106	0.541507	-0.009339
15	1	0	-4.008664	-0.600093	0.768483
16	8	0	-2.414378	2.035904	-0.019606
17	1	0	-2.524303	2.589229	-0.800649
18	1	0	-1.497376	1.692254	-0.047921

(HO)$_2$PO(.) 5H_2O
File = oh2po5c
E(UHF) = -950.5714739
ΔG = -950.465919

Center Number	Atomic Number	Atomic Type	Coordinates (Angstroms) X	Y	Z
1	15	0	-0.033371	0.288876	0.465447
2	8	0	-0.601645	-1.012599	-0.025152
3	8	0	-0.758423	1.589150	-0.036951
4	1	0	-1.778676	1.565419	0.003370
5	8	0	1.455057	0.577000	0.020075
6	1	0	2.099287	-0.220384	0.094777
7	8	0	-3.314376	1.459042	-0.043506
8	1	0	-3.510321	0.491131	-0.096720
9	1	0	-3.768915	1.797709	0.736304
10	1	0	-2.390028	-1.302529	-0.181285
11	8	0	-3.354814	-1.209518	-0.318531
12	1	0	-3.779464	-1.777372	0.333817
13	8	0	3.006319	-1.432878	0.096463
14	1	0	2.411478	-2.218765	0.001587
15	1	0	3.520166	-1.558872	0.902431
16	8	0	1.049824	-3.233736	-0.173013
17	1	0	0.969528	-3.710509	-1.006477
18	1	0	0.347320	-2.551751	-0.172845

19	8	0	2.695897	3.161184	-0.478332
20	1	0	2.704139	3.649282	0.351391
21	1	0	2.253335	2.322341	-0.276496

$(HO)_2PO(.)\ 6H_2O$

File = oh2po6a

E(UHF) = -1027.02072983

ΔG = -1026.893635

Center Number	Atomic Number	Atomic Type	Coordinates (Angstroms) X	Y	Z
1	15	0	-0.127739	0.006506	-0.473636
2	8	0	-1.552313	-0.117522	-0.017737
3	8	0	0.836916	-1.137733	0.035549
4	1	0	0.451197	-2.091639	-0.029224
5	8	0	0.626219	1.302382	0.012013
6	1	0	0.070360	2.169454	-0.013352
7	8	0	-0.156610	-3.470402	-0.027901
8	1	0	-1.137470	-3.338053	0.005964
9	1	0	0.033444	-3.991257	-0.816831
10	1	0	-2.449031	-1.698498	0.081622
11	8	0	-2.687479	-2.640486	0.197860
12	1	0	-3.345159	-2.841836	-0.476983
13	8	0	-0.776609	3.415355	0.054297
14	1	0	-1.715005	3.110566	0.138131
15	1	0	-0.728461	3.984067	-0.722864
16	8	0	-3.122342	2.154271	0.271912
17	1	0	-3.590558	2.186800	1.113481
18	1	0	-2.707759	1.269553	0.217580
19	1	0	3.963580	-1.366087	1.251178
20	8	0	3.708409	-1.066311	0.371786
21	1	0	2.745304	-1.183220	0.320937
22	8	0	3.609950	1.706684	-0.134633
23	1	0	2.644470	1.752378	-0.136682
24	1	0	3.792043	0.770270	0.066423

$(HO)_2PO(.)\ 7H_2O$

File = oh2po7a

E(UHF) = -1103.47196152

ΔG = -1103.324781

Center Number	Atomic Number	Atomic Type	Coordinates (Angstroms) X	Y	Z
1	15	0	-0.253392	-0.137377	-0.499491
2	8	0	0.553357	-1.244577	0.114225
3	8	0	0.069443	1.317292	0.013336
4	1	0	1.008560	1.703001	-0.208593
5	8	0	-1.802866	-0.231203	-0.223187
6	1	0	-2.200121	-1.178359	-0.221307
7	8	0	2.293238	2.371123	-0.453134
8	1	0	3.102202	1.814149	-0.282230
9	1	0	2.380588	2.737004	-1.340657
10	1	0	2.314055	-1.498656	0.161019
11	8	0	3.282766	-1.615457	0.249762
12	1	0	3.537507	-2.267804	-0.411759
13	8	0	-2.790800	-2.571457	-0.113051
14	1	0	-2.036648	-3.177123	0.097177
15	1	0	-3.205142	-2.900599	-0.918982
16	8	0	-0.485707	-3.798649	0.444529
17	1	0	-0.345906	-4.147176	1.332001
18	1	0	0.011908	-2.957603	0.394532
19	1	0	-1.736449	3.412270	1.940327
20	8	0	-1.726751	3.351745	0.978869

21	1	0	-1.064726	2.674221	0.761425
22	8	0	-3.821468	1.990792	-0.327537
23	1	0	-3.313788	1.175371	-0.435136
24	1	0	-3.211503	2.568629	0.166851
25	8	0	4.382453	0.826436	0.008496
26	1	0	4.044678	-0.095890	0.115599
27	1	0	4.886339	1.030857	0.803629

$(HO)_2PO(.)\ 8H_2O$

File = oh2po8c

E(UHF) = -1179.92252488

ΔG = -1179.753736

Center Number	Atomic Number	Atomic Type	Coordinates (Angstroms) X	Y	Z
1	15	0	0.594819	-0.009987	0.335599
2	8	0	0.737533	-1.439578	-0.146367
3	8	0	-0.486578	0.847531	-0.317791
4	1	0	-1.901551	0.587403	-0.256727
5	8	0	1.963356	0.801874	0.145775
6	1	0	2.796809	0.284125	0.378236
7	8	0	-2.938971	0.454771	-0.266649
8	1	0	-3.181052	-0.525075	-0.495285
9	1	0	-3.344770	0.728578	0.610543
10	1	0	-0.504210	-2.593554	-0.391636
11	8	0	-1.213897	-3.251724	-0.583922
12	1	0	-1.172767	-3.910721	0.117519
13	8	0	4.142022	-0.540150	0.643893
14	1	0	3.888706	-1.485477	0.508246
15	1	0	4.497630	-0.470176	1.536858
16	8	0	3.002893	-2.920101	0.170062
17	1	0	3.230457	-3.384329	-0.642723
18	1	0	2.139951	-2.476715	0.007573
19	1	0	-0.383771	3.408110	-2.257757
20	8	0	-0.322152	3.432475	-1.297035
21	1	0	-0.390711	2.501806	-0.999084
22	8	0	2.219838	3.725319	-0.136373
23	1	0	2.290029	2.778955	0.056191
24	1	0	1.361417	3.790263	-0.595006
25	8	0	-3.489800	-1.939078	-0.802125
26	1	0	-2.660688	-2.488945	-0.737674
27	1	0	-3.861299	-2.082766	-1.679997
28	8	0	-3.974172	1.241714	1.986004
29	1	0	-3.970179	0.604895	2.711225
30	1	0	-4.876864	1.579004	1.931741

$(HO)_2PO(.)\ 9H_2O$

File = oh2po9c

E(UHF) = -1256.37637725

ΔG = -1256.182805

Center Number	Atomic Number	Atomic Type	Coordinates (Angstroms) X	Y	Z
1	15	0	0.972104	-0.069409	0.250765
2	8	0	0.802944	-1.467829	-0.307620
3	8	0	0.210340	1.059131	-0.442522
4	1	0	-1.208202	1.141130	-0.542972
5	8	0	2.509638	0.379155	0.236018
6	1	0	3.161520	-0.336374	0.520452
7	8	0	-2.246671	1.260204	-0.663352
8	1	0	-2.668040	0.384085	-0.942364
9	1	0	-2.697906	1.535242	0.213043
10	1	0	-0.657831	-2.295814	-0.627689
11	8	0	-1.489689	-2.773910	-0.861328
12	1	0	-1.627115	-3.436133	-0.175107
13	8	0	4.225525	-1.475955	0.865758

14	1	0	3.772974	-2.320242	0.623597
15	1	0	4.469523	-1.544233	1.795559
16	8	0	2.618769	-3.467871	0.070915
17	1	0	2.820629	-3.917760	-0.756595
18	1	0	1.905865	-2.820556	-0.128603
19	1	0	1.140973	3.672985	-2.072142
20	8	0	1.085840	3.602443	-1.113282
21	1	0	0.767846	2.694902	-0.927791
22	8	0	3.458905	3.170644	0.327387
23	1	0	3.285868	2.221718	0.412292
24	1	0	2.704374	3.481876	-0.206659
25	8	0	-3.398483	-1.008945	-1.139148
26	1	0	-2.711555	-1.733565	-1.053379
27	1	0	-3.840526	-1.126945	-1.988457
28	8	0	-3.499149	1.724094	1.506561
29	1	0	-4.120302	0.954639	1.513453
30	1	0	-4.036475	2.523836	1.541840
31	8	0	-4.867032	-0.573751	1.215187
32	1	0	-4.504221	-0.859209	0.353808
33	1	0	-5.826439	-0.597718	1.129660

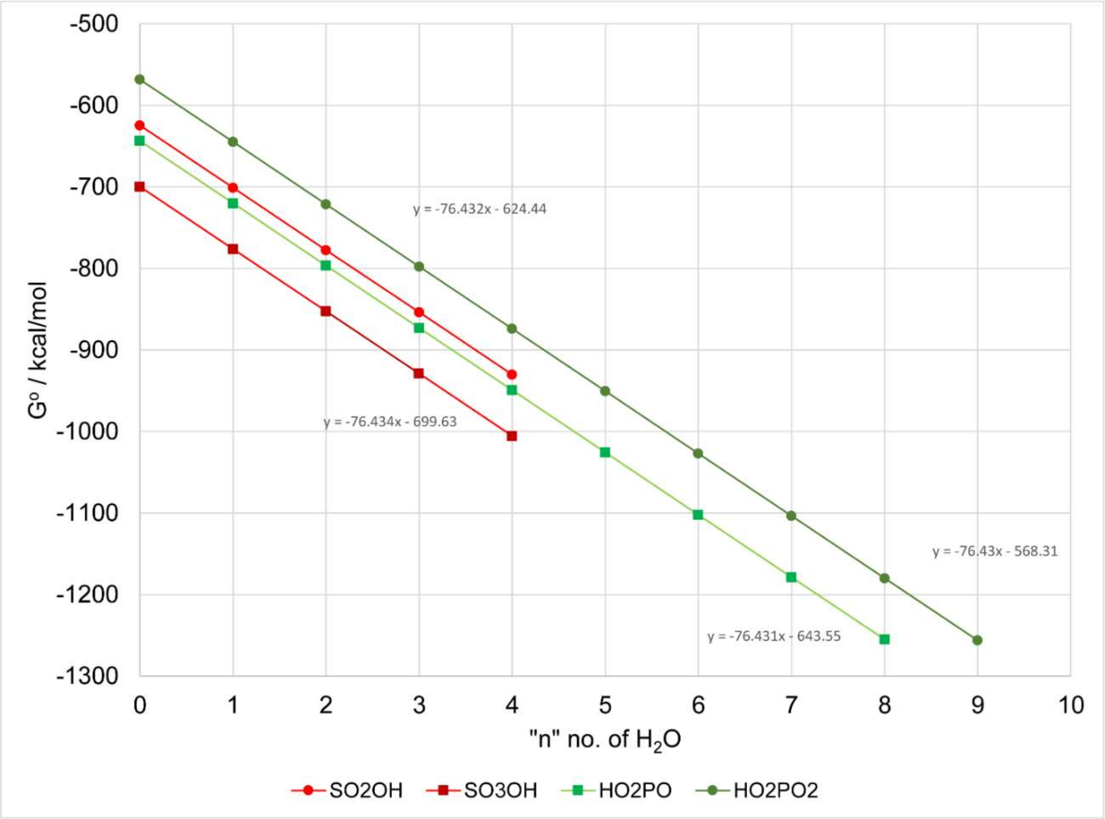

Figure S1. Plot of Gibbs Free Energies for microhydration clusters of S-radicals **4b**, **7** and P-radicals **10a, 13**, against no. of waters 'n' required for spontaneous ionization.

Transition States for Addition Reactions to Propene
CAM-B3LYP'6-311+G(2d,p) vacuum

•SO_2OH radical/propene TS 4b
File: o3sprTS1
E(UHF) = -742.28870205
ΔG = -742.221540
ν(imag.) = -250.09 cm^{-1}

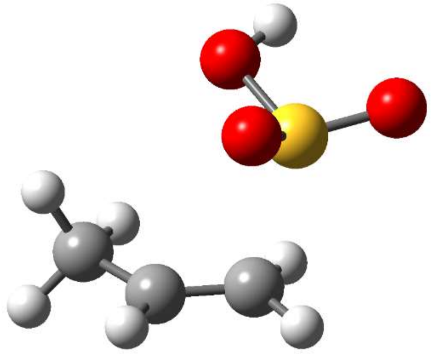

Center Number	Atomic Number	Atomic Type	Coordinates (Angstroms) X	Y	Z
1	16	0	0.992582	-0.083545	0.058640
2	8	0	0.607043	1.495804	-0.152080
3	1	0	1.061394	1.793330	-0.954343
4	8	0	1.128606	-0.262249	1.480175
5	8	0	2.093266	-0.348703	-0.848814
6	6	0	-1.069388	-1.119839	-0.640271
7	1	0	-0.970537	-0.723665	-1.644487
8	6	0	-2.001224	-0.614424	0.209521
9	1	0	-2.157973	-1.118326	1.157719
10	6	0	-2.752266	0.646058	-0.016220
11	1	0	-3.825689	0.485271	0.113064
12	1	0	-2.456968	1.401111	0.718730
13	1	0	-2.575682	1.052624	-1.011562
14	1	0	-0.649906	-2.103212	-0.469781

•PO(OH)$_2$ Radical/Propene TS (10b)
File: oh2popeTS1
E(UHF) = -686.16214007
ΔG° = -686.086237
ν(imag.) = -183.77 cm^{-1}

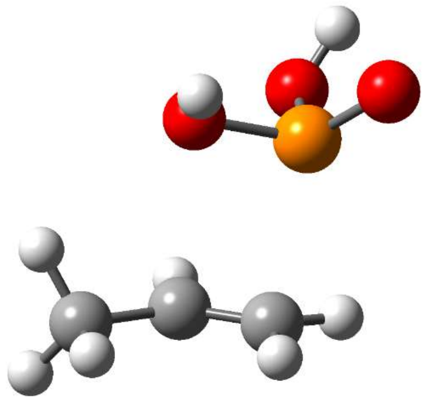

Center Number	Atomic Number	Atomic Type	Coordinates (Angstroms) X	Y	Z
1	15	0	-1.041263	-0.020637	-0.155303
2	8	0	-2.392108	0.033608	-0.757063
3	8	0	-0.999911	-0.786946	1.265990
4	1	0	-1.898401	-0.944295	1.587575
5	8	0	-0.483087	1.450376	0.243356
6	1	0	-1.003682	2.128902	-0.206569
7	6	0	1.262160	-1.058330	-0.865379
8	1	0	1.242729	-0.564456	-1.829849
9	1	0	0.813528	-2.041438	-0.812590
10	6	0	2.036434	-0.596668	0.131154
11	1	0	2.087658	-1.168279	1.052619
12	6	0	2.778057	0.692938	0.101485
13	1	0	2.707136	1.175236	-0.873436
14	1	0	3.833917	0.539610	0.338676
15	1	0	2.377008	1.380330	0.851297

Addition of $HOSO_3$• to propene

File = so4prTS2

E(UHF) = -817.4978297

ΔG^o = -817.4244

ν(imag.) = -164.1 cm^{-1}

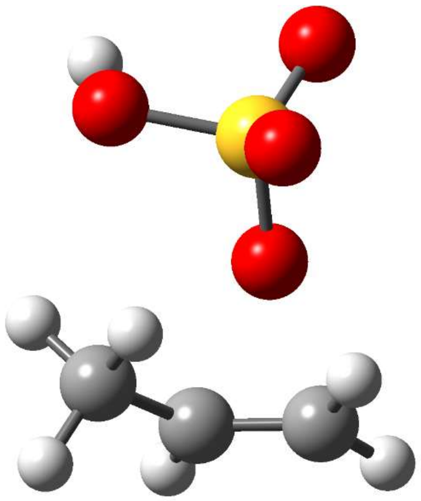

Center Number	Atomic Number	Atomic Type	Coordinates (Angstroms) X	Y	Z
1	6	0	-2.127030	-0.114122	-0.471449
2	1	0	-2.330685	-0.139544	-1.535752
3	6	0	-2.149820	1.208743	0.188035
4	1	0	-3.163752	1.617037	0.120785
5	1	0	-1.850618	1.150554	1.231719
6	1	0	-1.491961	1.905259	-0.333954
7	6	0	-1.984184	-1.304689	0.181133
8	1	0	-2.058613	-2.241925	-0.351433
9	1	0	-1.756673	-1.329396	1.238447
10	8	0	0.014237	-0.700713	-0.873061
11	8	0	0.543367	-0.067022	1.428941
12	16	0	1.038345	-0.097080	0.086657
13	8	0	2.325768	-0.650563	-0.215717
14	8	0	1.095888	1.457296	-0.352905
15	1	0	1.770895	1.539716	-1.040694

$(OH)_2PO_2$• Radical/Propene TS (13)

File: ho2po2peTS5

E(UHF) = -761.41286359

ΔG^o = -761.330544

ν(imag.) = -174.65 cm^{-1}

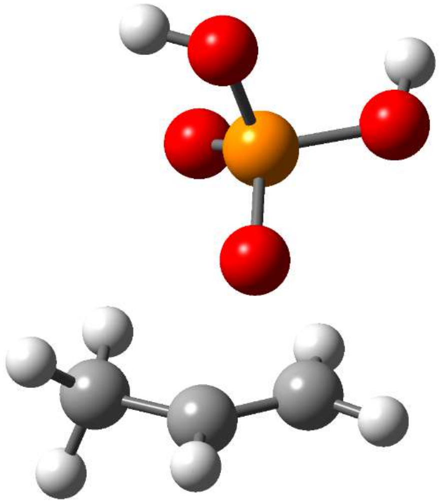

Center Number	Atomic Number	Atomic Type	Coordinates (Angstroms) X	Y	Z
1	15	0	-0.958549	0.093413	0.041733
2	8	0	0.079352	-0.267609	-1.054638
3	8	0	-1.663287	-1.303940	0.369415
4	1	0	-2.252601	-1.232771	1.129066
5	8	0	-2.121057	0.864837	-0.748211
6	1	0	-2.038611	1.817931	-0.630212
7	8	0	-0.501093	0.824406	1.233434
8	6	0	1.713520	-1.331581	0.196354
9	1	0	1.384903	-1.264271	1.225546
10	1	0	1.605457	-2.276253	-0.315867
11	6	0	2.253922	-0.251519	-0.428162
12	1	0	2.568162	-0.369330	-1.458967
13	6	0	2.522188	1.052024	0.218128
14	1	0	2.242334	1.869419	-0.447534
15	1	0	1.980665	1.155139	1.155482
16	1	0	3.598823	1.143839	0.398563

$HOCO_2^{\bullet}$ Radical/Propene TS
File: hoco2prTS1
E(UHF) =-382.20439585
ΔG^o = -382.130322
ν(imag.) = -109.43 cm^{-1}

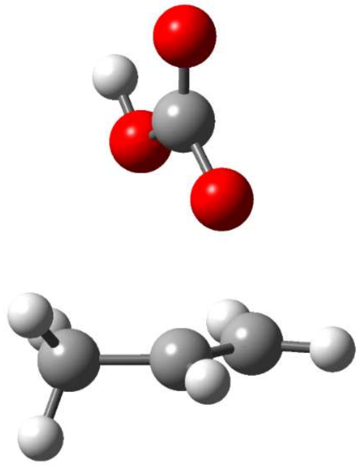

$HO^{\bullet}$ Radical/Propene TS
File: hoprTS1
E(UHF) = -193.61239991
ΔG^o = -193.550114
ν(imag.) = -77.16 cm^{-1}

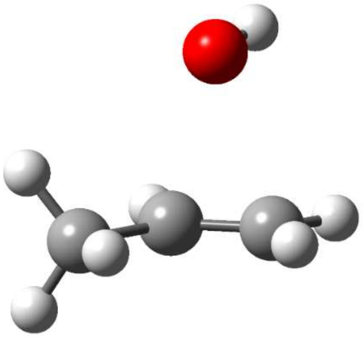

Center Number	Atomic Number	Atomic Type	Coordinates (Angstroms) X	Y	Z
1	6	0	-0.332954	1.211180	0.232048
2	1	0	-0.315814	1.232502	1.313714
3	1	0	-1.006337	1.896571	-0.266392
4	6	0	0.467433	0.392709	-0.458972
5	1	0	0.424183	0.415628	-1.543866
6	6	0	1.461448	-0.535470	0.145275
7	1	0	1.408321	-0.519759	1.233313
8	1	0	2.475159	-0.263046	-0.160510
9	1	0	1.278317	-1.555828	-0.193992
10	8	0	-1.470507	-0.888669	0.084807
11	1	0	-2.075333	-0.507232	-0.570830

Transition States for Abstraction Reactions from Propene
CAM-B3LYP'6-311+G(2d,p) vacuum

•SO_2OH radical 4b: TS for abstraction from propene
File: o3sabTS1
E(UHF) = -742.2617009
ΔG = -742.20262
ν(imag.) = -1565.14 cm^{-1}

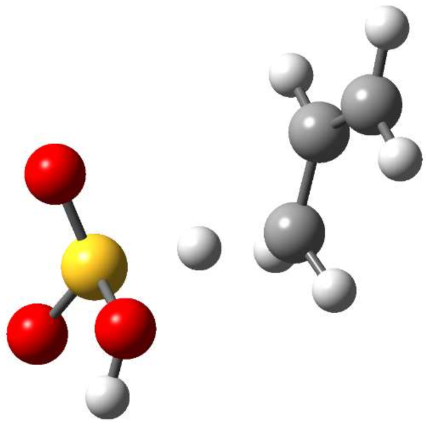

Center Number	Atomic Number	Atomic Type	Coordinates (Angstroms) X	Y	Z
1	16	0	1.126363	0.013499	0.152472
2	8	0	1.355858	0.719865	-1.293801
3	1	0	2.002840	0.185609	-1.778147
4	8	0	1.017967	1.072646	1.113505
5	8	0	2.127109	-1.021294	0.263624
6	6	0	-1.578499	-1.165954	-0.285146
7	1	0	-1.582106	-1.146191	-1.371555
8	6	0	-2.496444	-0.300703	0.390921
9	1	0	-2.640516	-0.461075	1.454453
10	1	0	-1.376562	-2.139372	0.151267
11	6	0	-3.142860	0.716686	-0.203785
12	1	0	-3.016925	0.921837	-1.260390
13	1	0	-3.810401	1.361565	0.351144
14	1	0	-0.298787	-0.608275	-0.064880

•$PO(OH)_2$ Radical (10b) TS for abstraction from Propene
File: oh2poabTS1
E(UHF) = -686.1408611
ΔG° = -686.072491
ν(imag.) = -1689.3 cm^{-1}

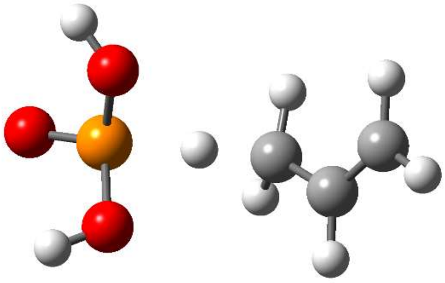

Center Number	Atomic Number	Atomic Type	Coordinates (Angstroms) X	Y	Z
1	15	0	-1.173389	-0.079130	-0.056964
2	8	0	-2.334318	-0.866480	-0.502990
3	8	0	-1.041147	0.136111	1.525932
4	1	0	-1.896952	0.112534	1.975826
5	8	0	-1.187805	1.434926	-0.616500
6	1	0	-1.785236	1.511907	-1.371801
7	6	0	1.639442	-1.073260	-0.551070
8	1	0	1.742253	-0.874531	-1.615309
9	1	0	1.484520	-2.124524	-0.324097
10	6	0	2.535556	-0.342666	0.322823
11	1	0	2.596740	-0.678367	1.353398
12	6	0	3.241826	0.727792	-0.048110
13	1	0	3.205175	1.103400	-1.064290
14	1	0	3.879125	1.253346	0.650356
15	1	0	0.380431	-0.624459	-0.343015

$HOSO_3$• Radical (7) TS for abstraction from Propene

File: hoso3peTS5

E(UHF) = -817.48484291

ΔG° = -817.415740

ν(imag.) = -601.57 cm^{-1}

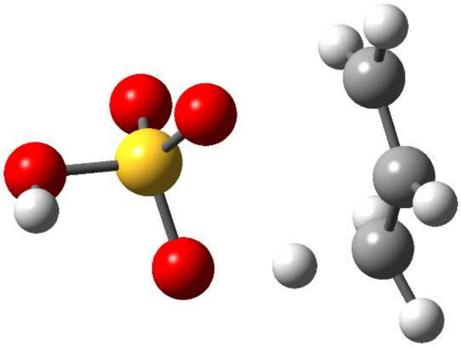

Center Number	Atomic Number	Atomic Type	Coordinates (Angstroms) X	Y	Z
1	8	0	-0.271957	1.045240	0.706956
2	8	0	-2.477022	0.003061	0.448470
3	16	0	-0.964248	0.004773	-0.066211
4	8	0	-1.045374	0.226480	-1.477593
5	8	0	-0.448926	-1.341176	0.320286
6	1	0	-2.477221	-0.129462	1.406939
7	6	0	2.203901	-1.161887	-0.189015
8	1	0	2.129765	-1.192115	-1.274676

9	1	0	1.033868	-1.329552	0.119873
10	1	0	2.748715	-1.993442	0.247858
11	6	0	2.411353	0.115696	0.390628
12	1	0	2.799336	0.170607	1.402471
13	6	0	1.971134	1.282119	-0.193147
14	1	0	1.551868	1.281696	-1.190867
15	1	0	2.069534	2.231489	0.312024

$(HO)_2PO_2$• Radical (13) TS for abstraction from Propene.

File: ho2po2peTS4

E(UHF) = -761.3964572

ΔG^o = -761.319579

ν(imag.) = -297.42 cm^{-1}

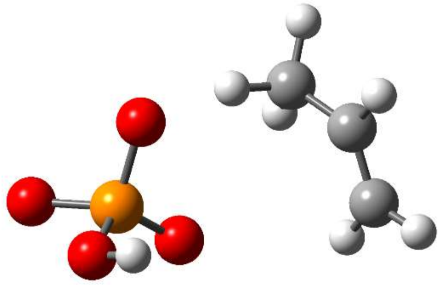

Center Number	Atomic Number	Atomic Type	Coordinates (Angstroms) X	Y	Z
1	15	0	-1.208091	0.060930	0.048785
2	8	0	-0.472742	-0.901547	-0.948185
3	8	0	-2.534359	-0.638841	0.555638
4	1	0	-2.431112	-1.008681	1.440030
5	8	0	-1.882532	1.155108	-0.877834
6	1	0	-1.250911	1.585969	-1.465828
7	8	0	-0.307970	0.513208	1.117550
8	6	0	2.164630	-1.229566	0.051547
9	1	0	1.907997	-1.221379	1.109463
10	1	0	2.654767	-2.163699	-0.227060
11	6	0	2.955013	1.101954	0.335198
12	1	0	2.407879	1.203576	1.264402
13	6	0	2.923496	-0.033534	-0.358583
14	1	0	3.519021	1.958316	-0.012567
15	1	0	3.460794	-0.089389	-1.301123
16	1	0	1.174927	-1.235211	-0.485425

HO(.) Radical TS for Abstraction from Propene

File: HOpeTS1

E(UHF) = -193.60509232

ΔG = -193.547317

ν(imag) = -77.16 cm^{-1}

Center Number	Atomic Number	Atomic Type	Coordinates (Angstroms) X	Y	Z
1	6	0	0.100999	0.948957	0.147739
2	1	0	-0.050622	1.117929	1.212901
3	1	0	0.235613	1.898388	-0.372970
4	6	0	-1.889512	-0.535434	0.220946
5	1	0	-1.959885	-0.451823	1.299889
6	6	0	-0.945238	0.097733	-0.460631
7	1	0	-2.630175	-1.149703	-0.274700
8	1	0	-0.911527	-0.011739	-1.540809
9	1	0	1.129382	0.448867	0.040104

10	8	0	2.340900	-0.456283	-0.005548
11	1	0	1.862522	-1.269192	0.231647

$HOCO_2$(.) Radical TS for abstraction from Propene

File: hoco2prTS1

E(UHF) = -382.20439585

ΔG = -382.130322

ν(imag) = -109.43 cm^{-1}

Center Number	Atomic Number	Atomic Type	Coordinates (Angstroms) X	Y	Z
1	8	0	-0.465994	0.785085	0.625820
2	8	0	-0.934712	-0.739705	-0.936122
3	1	0	-1.709741	-1.230267	-1.242284
4	8	0	-2.531934	0.061559	0.424578
5	6	0	1.550516	0.985148	-0.845251
6	1	0	1.403158	0.436907	-1.766466
7	1	0	1.516716	2.064837	-0.886426
8	6	0	1.722315	0.339795	0.328371
9	1	0	1.876892	0.933828	1.221240
10	6	0	1.872593	-1.130924	0.475489
11	1	0	1.247848	-1.501049	1.289622
12	1	0	1.612772	-1.657939	-0.440135
13	1	0	2.908469	-1.364825	0.738316
14	6	0	-1.377923	0.049814	0.070046

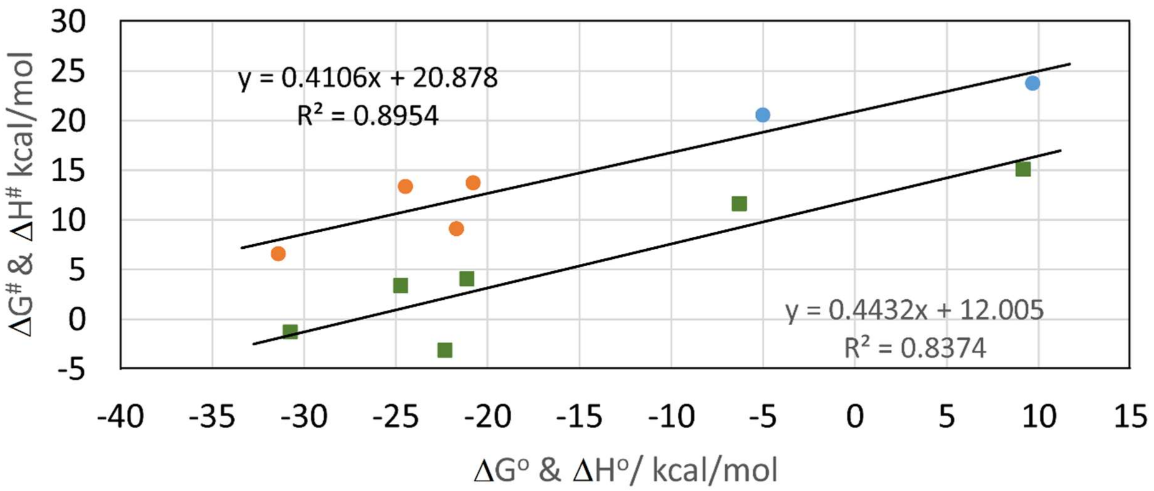

Figure S2. Plots of activation enthalpy ($\Delta H^{\#}$) vs. enthalpy of reaction (ΔH^{o}, squares) of the H-abstraction reactions from propene and of the corresponding Gibbs free energies ($\Delta G^{\#}$ vs. ΔG^{o}, circles).

Table S1. Computed energetics for addition and H-atom abstraction reactions of S-, P- and model radicals with propene showing results with pentanoic acid as solvent[a]

	**Addition C-1**		**Allylic H-Abstraction**		**Addition C-2**
**Radical**	**ΔG°**	**ΔG‡**	**ΔG°**	**ΔG‡**	**ΔG°**
$^{\bullet}SO_2OH$, **4b**	2.3	11.8	9.7	23.7	5.5
$HOSO_3^{\bullet}$, **7**	-12.7	3.6	-21.7	9.1	-9.2
$^{\bullet}PO(OH)_2$, **10b**	-13.0 [-11.5]	11.9	-5.0 [-5.2]	20.5	-7.9 [-7.0]
$(HO)_2PO_2^{\bullet}$, **13**	-14.9 [-14.2]	6.5	-24.5 [-26.4]	13.3	-11.9 [-10.2]
$(HO)CO_2^{\bullet}$	-9.8	10.0	-20.8	5.7	
$HO^{\bullet}$	-19.9	4.8	-31.4 [-32.3]	6.6	

[a] Free energies (kcal mol^{-1}) computed at the CAM-B3LYP/6-311+G(2d,p)//CAM-B3LYP/6-311 +G(2d,p) level in vacuo except for those with pentanoic acid [in square brackets] which were calculated at the CAM-B3LYP/6-311+G(2d,p)//CAM-B3LYP/6-31G(2d,p) level with the CPCM continuum and pentanoic acid as solvent.

Table S2. Computed potential energies for addition and H-atom abstraction reactions of S-, P- and model radicals with propene[a]

	**Addition C-1**		**Allylic H-Abstraction**	
**Radical**	**ΔE°**	**ΔE‡**	**ΔE°**	**ΔE‡**
$^{\bullet}SO_2OH$, **4b**	-9.16	1.53	9.18	15.07
$HOSO_3^{\bullet}$, **7**	-23.13	-8.21	-22.32	-3.16
$^{\bullet}PO(OH)_2$, **10b**	-23.81	1.64	-6.29	11.57
$(HO)_2PO_2^{\bullet}$, **13**	-25.21	-4.97	-24.71	3.33
$(HO)CO_2^{\bullet}$	-20.35	-0.98	-21.13	4.01
$HO^{\bullet}$	-27.76	-3.23	-30.73	-1.34

[a] Zero-point corrected potential energies (ΔE + ZPE, kcal mol^{-1}) computed at the CAM-B3LYP/6-311+G(2d,p)//CAM-B3LYP/6-311 +G(2d,p) level in vacuo.

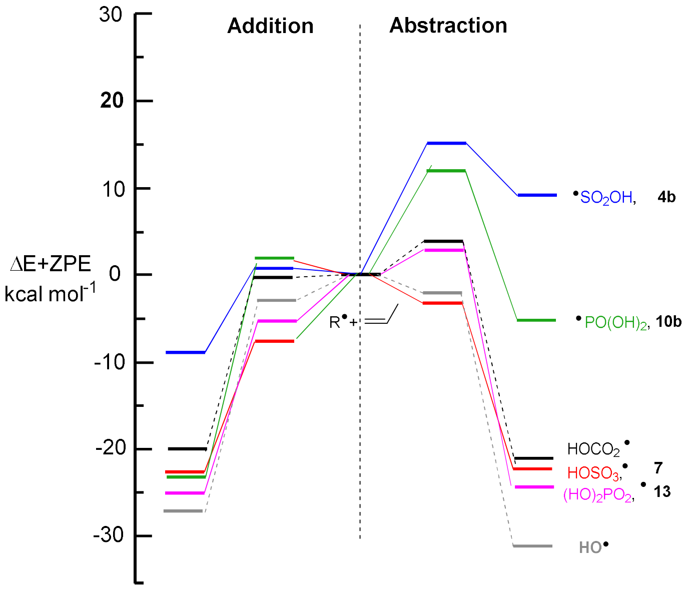

Figure S3. Schematic of the reaction coordinates for addition and abstraction from propene of S- and P-based radicals and model radicals (zero-point corrected potential energy data from Table S2. Blue lines: $^{\bullet}SO_2OH$ radicals **4b**. Green lines: $^{\bullet}PO(OH)_2$ radicals **10b**. Black lines, $HCO_2^{\bullet}$ radicals. Red lines, $HOSO_3^{\bullet}$ radicals **7**. Purple lines, $(HO)_2PO_2^{\bullet}$ radicals **13**. Grey lines, $HO^{\bullet}$ radicals.

References

(1) Gaussian 09, Revision D.01; Gaussian, Inc.: Wallingford, CT, 2013. M. J. Frisch, G. W. Trucks, H. B. Schlegel, G. E. Scuseria, M. A. Robb, J. R. Cheeseman, G. Scalmani, V. Barone, B. Mennucci, G. A. Petersson, H. Nakatsuji, M. Caricato, X. Li, H. P. Hratchian, A. F. Izmaylov, J. Bloino, G. Zheng, J. L. Sonnenberg, M. Hada, M. Ehara, K. Toyota, R. Fukuda, J. Hasegawa, M. Ishida, T. Nakajima, Y. Honda, O. Kitao, H. Nakai, T. Vreven, J. A. Montgomery, Jr., J. E. Peralta, F. Ogliaro, M. Bearpark, J. J. Heyd, E. Brothers, K. N. Kudin, V. N. Staroverov, R. Kobayashi, J. Normand, K. Raghavachari, A. Rendell, J. C. Burant, S. S. Iyengar, J. Tomasi, M. Cossi, N. Rega, J. M. Millam, M. Klene, J. E. Knox, J. B. Cross, V. Bakken, C. Adamo, J. Jaramillo, R. Gomperts, R. E. Stratmann, O. Yazyev, A. J. Austin, R. Cammi, C. Pomelli, J. W. Ochterski, R. L. Martin, K. Morokuma, V. G. Zakrzewski, G. A. Voth, P. Salvador, J. J. Dannenberg, S. Dapprich, A. D. Daniels, O. Farkas, J. B. Foresman, J. V. Ortiz, J. Cioslowski, and D. J. Fox.

(2). Curtiss, L. A.; Redfern, P. C.; Raghavachari, K. Gaussian-4 theory, *J. Chem. Phys.*, **2007**, *126*, 84108-84119.

(3). Armstrong, D. A.; Waltz, W. L.; Rauk, A. Carbonate radical anion – Thermochemistry, *Can. J. Chem.*, **2006**, *84*, 1614-1619.

(4). Yanai, T.; Tew, D. P.; Handy, N. C. A New Hybrid Exchange-correlation Functional Using the Coulomb-attenuating Method (CAM-B3LYP). *Chem. Phys. Lett*. **2004**, *393*, 51-57.

(5). Tawada, Y.; Tsuneda, T.; Yunagisawa, S.; Yanai, T.; Hirao, K. A Long-Range-Corrected Time-Dependent Density Functional Theory. *J. Chem. Phys.* **2004**, *120*, 8425-8433.

(6). (a) Hutson, T. Computational Investigation of the Bisulfate and Bisulfite Radicals and their Reactivities, MChem Dissertation, University of St. Andrews, 2019.
